# The alphavirus determinants of intercellular long extension formation

**DOI:** 10.1128/mbio.01986-24

**Published:** 2024-12-19

**Authors:** Caroline K. Martin, Judy J. Wan, Peiqi Yin, Thomas E. Morrison, William B. Messer, Vanessa Rivera-Amill, Jonathan R. Lai, Nina Grau, Félix A. Rey, Thérèse Couderc, Marc Lecuit, Margaret Kielian

**Affiliations:** 1 Department of Cell Biology, Albert Einstein College of Medicine, Bronx, New York, USA; 2 Department of Immunology and Microbiology, University of Colorado School of Medicine, Aurora, Colorado, USA; 3 Department of Molecular Microbiology and Immunology, Oregon Health & Science University, Portland, Oregon, USA; 4 Department of Medicine, Division of Infectious Diseases, Oregon Health & Science University, Portland, Oregon, USA; 5 Ponce Health Sciences University/Ponce Research Institute, Ponce, Puerto Rico; 6 Department of Biochemistry, Albert Einstein College of Medicine, Bronx, New York, USA; 7 Institut Pasteur, Université Paris Cité, CNRS UMR 3569, Unité de Virologie Structurale, Paris, France; 8 Institut Pasteur, Université Paris Cité, Inserm U1117, Biology of Infection Unit, Paris, France; 9 Department of Infectious Diseases and Tropical Medicine, Necker-Enfants Malades University Hospital, APHP, Institut Imagine, Paris, France; Griffith University-Gold Coast Campus, Gold Coast, Queensland, Australia; Purdue University, West Lafayette, Indiana, USA

**Keywords:** alphavirus, chikungunya, intercellular transmission, virus budding, virus exit

## Abstract

The alphavirus chikungunya virus (CHIKV) is a serious human pathogen that can cause large-scale epidemics characterized by fever and joint pain and often resulting in chronic arthritis. Infection by alphaviruses including CHIKV and the closely related Semliki Forest virus (SFV) can induce the formation of filopodia-like intercellular long extensions (ILEs). ILEs emanate from an infected cell, stably attach to a neighboring cell, and mediate cell-to-cell viral transmission that is resistant to neutralizing antibodies. However, our mechanistic understanding of ILE formation is limited, and the potential contribution of ILEs to CHIKV virulence or human CHIKV infection is unknown. Here, we used well-characterized virus mutants and monoclonal antibodies with known epitopes to dissect the virus requirements for ILE formation. Our results showed that both the viral E2 and E1 envelope proteins were required for ILE formation, while viral proteins 6K and transframe, and cytoplasmic nucleocapsid formation were dispensable. A subset of CHIKV monoclonal antibodies reduced ILE formation by masking specific regions particularly on the E2 A domain. Studies of the viral proteins from different CHIKV strains showed that ILE formation is conserved across the four major CHIKV lineages. Sera from convalescent human CHIKV patients inhibited ILE formation in cell culture, providing the first evidence for ILE inhibitory antibody production during human CHIKV infections.

**IMPORTANCE:**

Chikungunya virus (CHIKV) infections can cause severe fever and long-lasting joint pain in humans. CHIKV is disseminated by mosquitoes and is now found world-wide, including in the Americas, Asia, and Africa. In cultured cells, CHIKV can induce the formation of long intercellular extensions that can transmit virus to another cell. However, our understanding of the formation of extensions and their importance in human CHIKV infection is limited. We here identified viral protein requirements for extension formation. We demonstrated that specific monoclonal antibodies against the virus envelope proteins or sera from human CHIKV patients can inhibit extension formation. Our data highlight the importance of evaluation of extension formation in the context of human CHIKV infection.

## INTRODUCTION

Alphaviruses are positive-sense, single-stranded RNA viruses that can cause severe human and animal illnesses, including life-threatening encephalitis and persistent arthritis (1, 2). Chikungunya virus (CHIKV) is a member of the arthritogenic alphaviruses. CHIKV infection in humans causes fever and rash accompanied by severe muscle and joint pain that can persist for years (3–5), and can also cause lethal encephalitis (6). CHIKV is transmitted by *Aedes* mosquitoes and has spread across the globe. It is currently endemic in the Americas, Africa, and Asia, with the majority of the ~500,000 patients reported in 2023 located in Central and South America (European CDC, December, 2023) (7–12). Infections by CHIKV and other alphaviruses are expected to continue to rise globally due to the spread of their mosquito vectors and the adaption of the viruses to new vectors (8, 9). Despite this important health burden, there are currently no approved antivirals for treatment of any human alphavirus infection, and only a single FDA-licensed CHIKV vaccine (Ixchiq) (13).

The alphavirus genome encodes two open reading frames, one for the non-structural proteins (nsP1-4) that replicate the viral genome, and one for the structural proteins that form the virus particle: capsid protein (Cp), p62 (precursor of E3 and E2), 6K/transframe (TF), and the membrane fusion protein E1 (2, 14, 15). In the mature virion, the viral RNA genome is packaged in a shell of 240 Cp to form the core nucleocapsid (NC). During budding at the plasma membrane, the NC is enveloped in a host cell-derived lipid membrane that is studded with 80 viral spikes composed of trimers of E2-E1 dimers (16, 17). These spikes are anchored to the underlying NC through the binding of the cytoplasmic domain of E2 to a hydrophobic pocket in Cp, an interaction that is critical for virus budding (18). Alphavirus infection is initiated by the E2-E1 spikes, which mediate cell surface attachment and receptor engagement, thus promoting virus uptake by clathrin-mediated endocytosis (19–26). The endosomal low pH then triggers the E1 membrane fusion protein, which mediates viral-endosomal membrane fusion to release the NC into the host cell cytoplasm (2, 23, 27). The non-structural proteins are translated to form the viral RNA replication complex, and the structural proteins are expressed as a polyprotein. Cp autocatalytically cleaves itself from the nascent polyprotein chain, remains in the cytoplasm, and encapsulates the viral genome, thus forming the NC (28, 29). The rest of the structural proteins are translocated into the ER where p62 forms a stable heterodimer with E1. During transport through the secretory pathway, p62 is cleaved by the cellular protease furin into mature transmembrane E2 and peripheral E3. The glycoproteins are then delivered to the plasma membrane where virus particle assembly and budding occur (14). Virus budding requires the 1:1 interaction between Cp and the cytoplasmic endodomain of E2 (30, 31). Mutation of specific E2 residues, including a conserved tyrosine (SFV E2 Y399R; SINV E2 Y400K), can prevent Cp-E2 interaction and budding (18).

Several alphaviruses, including CHIKV and Semliki Forest virus (SFV), induce the formation of arm-like extensions that emanate from an infected cell and stably attach to a neighboring cell (32). These intercellular long extensions (ILEs) are at least 10 µm long, contain both actin and tubulin, and are closed-ended, without cytoplasmic or membrane continuity between the ILE and the target cell. ILEs can form in a variety of mammalian cell lines including Vero (African green monkey kidney), U-2 OS (human osteosarcoma), mouse embryonic fibroblasts (MEF), and primary human umbilical vein endothelial cells (HUVEC) although some cell line-specific differences have been observed (32, 33). In cell culture, ILEs have been shown to promote CHIKV cell-to-cell transmission that is resistant to high concentrations of a potent neutralizing monoclonal antibody (mAb chCHK-152) (33). *In vivo*, CHIKV infection by direct virus inoculation is blocked by pretreatment of mice with chCHK-152. In contrast, adoptive transfer of CHIKV-infected MEFs produced antibody-resistant virus infection, supporting a role for ILEs in cell-to-cell transmission *in vivo* (33).

Our current understanding of the mechanism of ILE formation is still limited, and the potential contribution of ILEs to CHIKV virulence and/or human CHIKV infection has not been addressed. Prior studies showed that the expression of the viral structural proteins is necessary and sufficient to induce the formation of ILEs, ruling out an essential role for the nsPs or virus infection per se (32). Although virus budding is dispensable for ILE formation, the viral Cp and its interaction with the E2 protein are essential (32), while the requirements for NC assembly and envelope protein expression are unclear. To address these gaps in knowledge, we used a combination of well-characterized virus mutants and specific anti-CHIKV mAbs. Our results showed that while the viral 6K and transframe proteins and cytoplasmic NC formation were dispensable for ILE formation, both the viral E2 and E1 proteins were involved, and specific mAbs to the envelope proteins could reduce ILE formation. We found that the ability to form ILEs was conserved across all four major CHIKV lineages, including the attenuated 181/25 strain. While the role of ILEs in human CHIKV infections remains unclear, we describe the first evidence that human CHIKV patients produce Abs that can significantly reduce the formation of ILEs by human cells in culture.

## RESULTS

### Cytoplasmic pre-assembly of the nucleocapsid is not essential for the formation of ILEs

**Fig 1:**
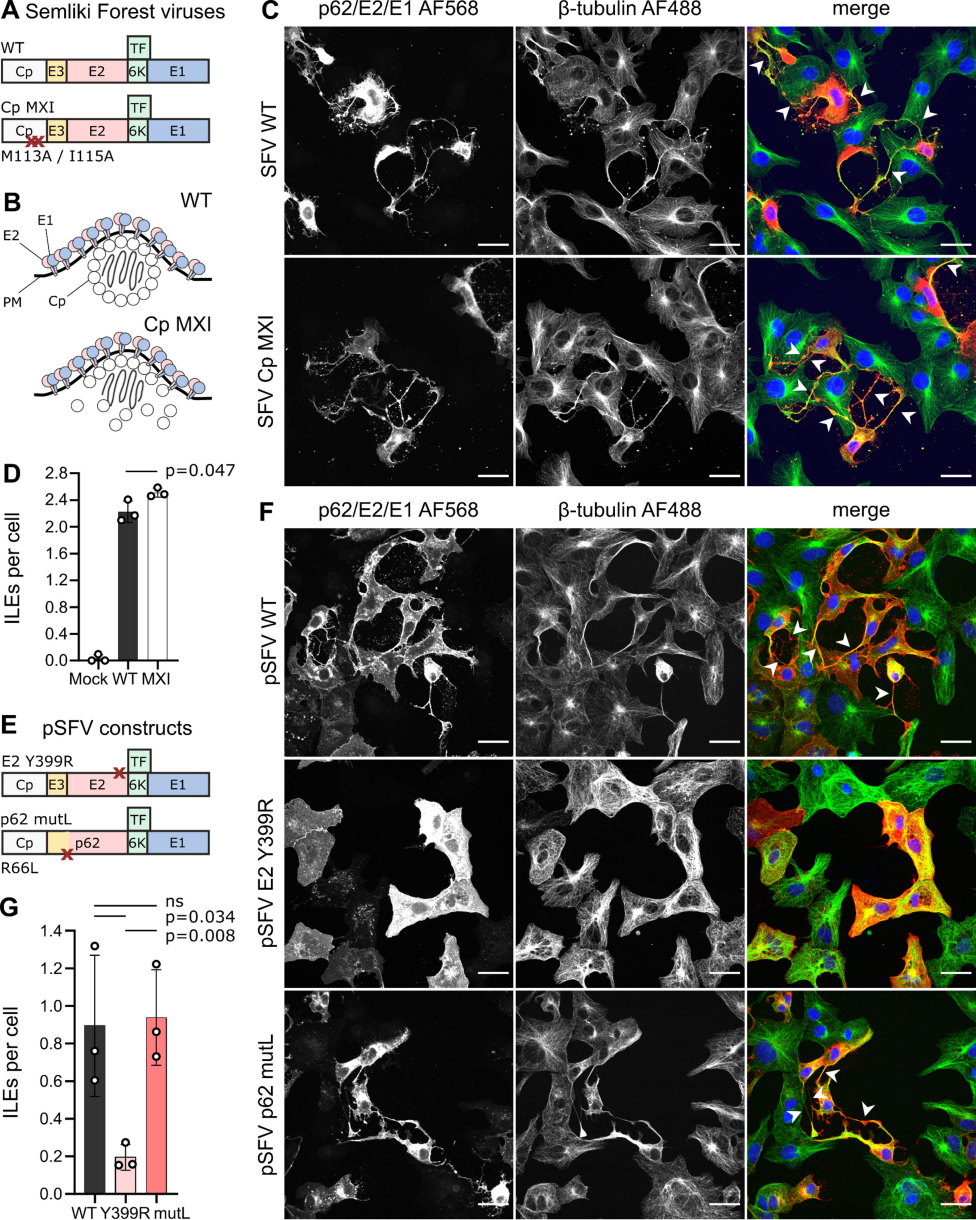
Cytoplasmic nucleocapsid pre-assembly and p62 processing are not required for ILE formation. (A) Schematic of the structural proteins for the WT and MXI mutant Semliki Forest viruses. Red Xs indicate the sites of the MXI mutations (M113A and I115A) that inhibit Cp-Cp interaction and cytoplasmic nucleocapsid pre-assembly. (B) Schematic of the differences in nucleocapsid assembly in SFV WT vs SFV Cp MXI. Shown are the E2-E1 dimers on the plasma membrane (PM) and the Cp proteins surrounding the viral RNA, either in a pre-assembled NC (WT) or *in situ* assembling NC (MXI). (C and D) Vero cells were infected with either WT SFV or the SFV Cp MXI mutant (MOI_Vero_ 0.30). Cells were fixed at 8 h post infection (hpi), stained for DNA (Hoechst, blue), and immuno-stained for the SFV envelope proteins (p62/E2-E1, red) and β-tubulin (green). Z-stacks were acquired by confocal microscopy, and the number of ILEs was manually quantitated. (E) Schematic of expression of the mutant SFV structural proteins (WT proteins as depicted in panel A). Red Xs indicate the sites of the Y399R mutation that blocks E2-Cp interaction and the mutL furin cleavage site mutation in p62 R66L. (F and G) Vero cells were transfected with the indicated plasmids expressing the WT, Y399R, or mutL SFV structural proteins. Samples were fixed at 24 h post transfection (hpt) and analyzed as described in panels C and D. (C, F) For each condition, a representative single slice micrograph is shown; arrow heads point out ILEs; scale bar = 30 µm. (D, G) Bar graphs show the means of three biological replicates (white circles) ±S.D. Significance was determined using non-parametric, unpaired, two-tailed student *t*-tests, with *P* values rounded to three decimal points. Differences were considered significant if *P* < 0.05. Total number of cells analyzed per condition: (D) mock (131), SFV WT (178), SFV Cp MXI (135); (G) pSFV WT (315), pSFV E2 Y399R (287), pSFV p62 mutL (236).

While Cp expression and its interaction with E2 are critical for ILE formation (32), the requirements for Cp-Cp interaction and NC cytoplasmic pre-assembly are unknown. To address this, we infected Vero cells with wild-type (WT) SFV or SFV MXI, an SFV mutant that contains alanine substitutions at Cp positions M(113) and I(115) (Fig. 1A) (34, 35). These mutations have been shown to destabilize Cp-Cp interactions and prevent cytoplasmic pre-assembly of the NC. Despite this NC assembly defect, the SFV MXI mutant is viable and produces infectious virus particles with only an ~10-fold reduced titer and normal NC architecture (35). Together the data support a model in which the assembly of Cp MXI into NC is driven by interactions with the envelope protein lattice at the plasma membrane to promote virus budding (35) (Fig. 1B, and see also (36)). We quantitated ILE formation at 8 h post infection (hpi) by confocal microscopy and found that SFV MXI infection induced a comparable number of ILEs per cell (mean number of ILEs per cell ±S.D.: 2.5 ± 0.1) as infection by the WT SFV control (2.2 ± 0.2) (Fig. 1B and C). Thus, neither cytoplasmic pre-assembly of the NC nor the Cp MXI motif were essential for ILE formation.

### Cleavage of p62 is not required for ILE formation

The p62 precursor is cleaved in the secretory pathway by the cellular protease furin into mature E2 and E3 (37, 38). To test if immature p62 could induce ILEs, we used the furin-insensitive SFV mutant L (mutL), which has a leucine substitution for an arginine in the furin cleavage site (39). SFV mutL buds immature virus particles that are strongly attenuated in virus fusion and infection (38, 40, 41). To circumvent the infection block, we evaluated ILE formation induced by plasmids that encode the wild-type (pSFV WT) or mutL (pSFV p62 mutL) SFV structural proteins (Fig. 1D). As a negative control, we expressed the SFV structural proteins containing an E2 Y399R mutation (pSFV E2 Y399R), which blocks E2-Cp interaction (18) and is defective in ILE formation (32). We transfected Vero cells with the pSFV constructs, cultured for 24 h, and scored ILE formation (Fig. 1E and F). Our results showed that WT and mutL had similar levels of ILE formation (WT 0.9 ± 0.4 and mutL 0.9 ± 0.3 ILEs per expressing cell). In keeping with prior results, the E2 Y399R mutant induced significantly fewer ILEs (0.2 ± 0.1).

### Neither 6K nor transframe are essential for ILE formation

The alphavirus structural proteins Cp-E3-E2-6K/TF-E1 are sufficient for ILE formation, and Cp and E2 are essential (32). However, the contribution of the small membrane proteins 6K and transframe (TF) are currently unknown. This is especially important since 6K was suggested to support E2/E1 spike maturation and Cp-E2 interaction (42, 43). 6K and TF share the same N-terminus but differ in their C-terminal amino acid sequence due to an internal frameshifting event that produces TF (reviewed in reference 43).

**Fig 2:**
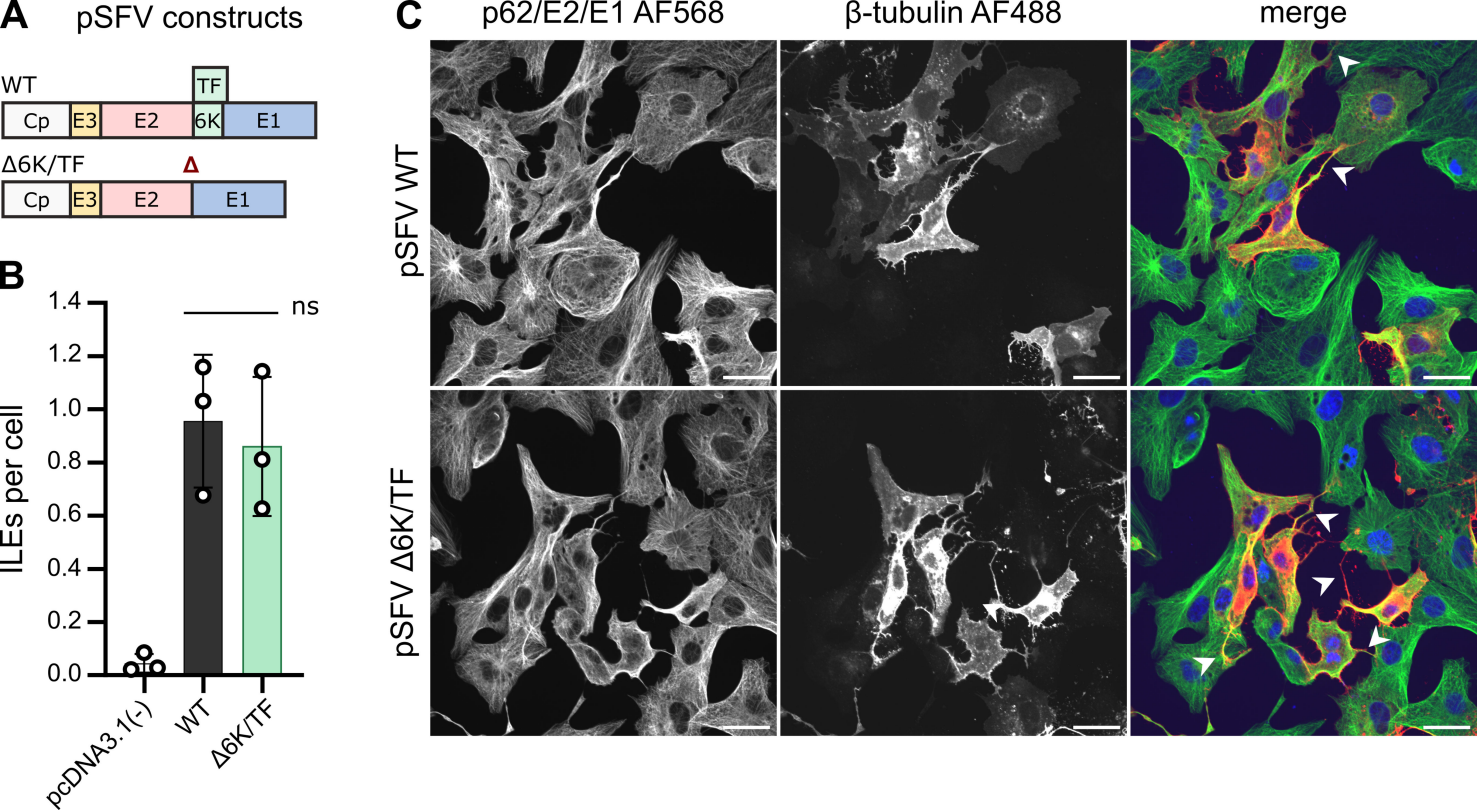
The viral proteins 6K and transframe (TF) are dispensable for the formation of ILEs. (A) Schematic of the SFV WT or 6K/TF deletion structural proteins. Red triangle indicates the 6K/TF deletion. (B and C) Vero cells were transfected with the indicated plasmids expressing the WT or Δ6K/TF SFV structural proteins, or the control plasmid pcDNA3.1(−). Samples were fixed at 24 h post transfection (hpt) and analyzed as described in Fig. 1. (B) Bar graph shows the means of three biological replicates (white circles) ±S.D. Significance was determined using non-parametric, unpaired, two-tailed student *t*-tests, with *P* values rounded to three decimal points. Differences were considered significant if *P* < 0.05. Total number of cells analyzed per condition: (B) pcDNA3.1(−) (212), pSFV WT (262), pSFV Δ6K/TF (315). (C) For each condition, a representative single slice micrograph is shown; arrow heads point out ILEs; scale bar = 30 µm.

While neither 6K nor TF are essential for infection in cell culture, the deletion of the *6K* sequence reduces virus budding, growth, and fusion capacity (43–45). To avoid these confounding effects of the 6K/TF deletion virus, we generated a plasmid that expresses the SFV WT structural proteins but lacks the entire 6K and TF coding region (pSFV Δ6K/TF) (Fig. 2A). We transfected Vero cells with either the pSFV Δ6K/TF, the pSFV WT construct, or a control plasmid (pcDNA3.1(−)), and quantitated ILEs at 24 hpt (Fig. 2B and C). Transfected cells formed similar numbers of ILEs per cell whether 6K and TF were expressed (pSFV WT: 1.0 ± 0.2), or not (pSFV Δ6K/TF: 0.9 ± 0.3), indicating that 6K and TF are dispensable for ILE formation. Given the reported contribution of 6K to virus budding and spike maturation (43), this result is in line with the published observation that ILE formation does not require virus budding (32). It further implies that 6K’s suggested contribution to Cp-E2 interaction is negligible for ILE formation.

### Antibody interaction with E2 domain A attenuates ILE formation

**Fig 3:**
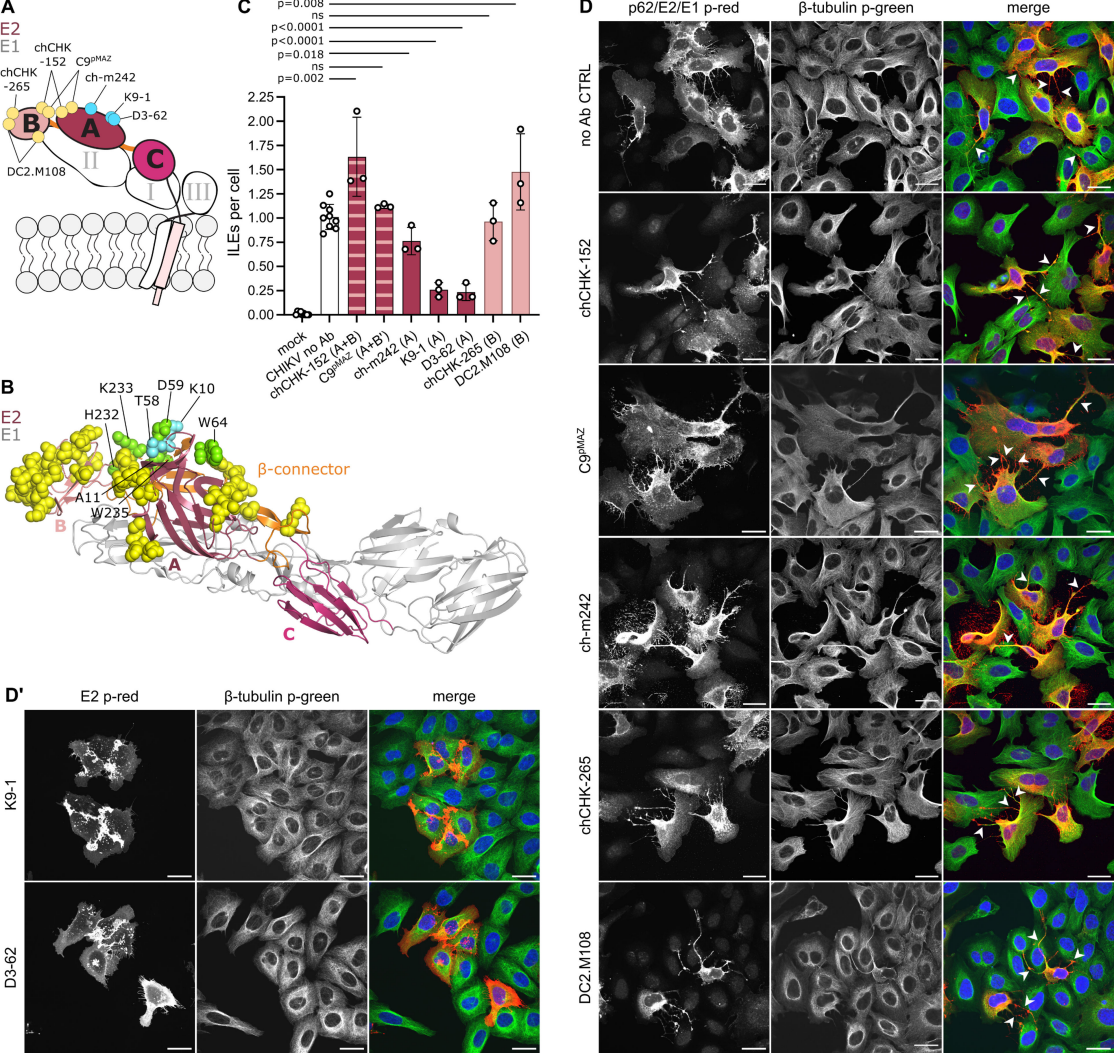
Masking of E2 domain A by mAb binding attenuates ILE formation. (A) Schematic representation of the CHIKV E2-E1 dimer showing E2 and its domains A, B, C, and β-ribbon connector in pink and E1 and its domains I, II, III in gray. Highlighted are the approximate binding sites of the CHIKV E2 mAbs, where yellow circles highlight mAbs that did not attenuate ILEs; blue circles indicate mAbs that did attenuate ILEs. (B) Crystal structure of the CHIKV E2-E1 dimer (PDB accession number 3J2W [49]); domains colored as in panel A. Critical epitope binding residues for tested mAbs are shown as spheres (as determined by neutralization escape mutants, mutational scanning, or structural studies, see Table S1): yellow residues for mAbs that did not reduce ILE formation; cyan residues for ILE-attenuating mAbs; green residues for those shared between ILE attenuating and non-attenuating mAbs. (C and D) Effect of anti-E2 mAbs on ILE formation. U-2 OS cells were infected with CHIKV 181/25 GFP (MOI_U-2 OS_ 0.25) for 2 h, then the inoculum was replaced with medium containing 20 µg/mL of the indicated mAb. Cells were cultured for an additional 9 h and then fixed and analyzed as in Fig. 1. (D′) As in panel D but immuno-stained for the SFV envelope protein E2 (p-red). (D-D′) For clarity, micrographs were pseudo-coloured to represent viral proteins in red and β-tubulin in green. For each condition, a representative single slice micrograph is shown; arrow heads point out ILEs; scale bar indicates 30 µm. (C) Bar graph shows the means of 3 or 9 biological replicates (white circles) ±S.D. Please note, subsets of mock and no Ab data were reproduced in Figure 5C, Figure 6B, Figure 8B, and Figure S5 as respective experiments were done in parallel. Significance was determined as in Fig. 1. Total number of analyzed cells per condition: mock (753), no Ab (774), chCHK-152 (226), C9^pMAZ^ (188), ch-m242 (313), K9-1 (271), D3-62 (319), chCHK-265 (218), DC2.M108 (239).

To understand the role of p62/E2 in ILE formation in more detail, we next focused on the contribution of individual E2 domains. Mature E2 contains an ectodomain that is subdivided into three domains: a central domain A, joined by a β-ribbon connector to domain B at the E2 tip and to the membrane proximal domain C, which is linked via subdomain D to the transmembrane (TM) anchor and endodomain (Fig. 3A and B) (46, 47). To dissect the importance of individual E2 domains, we tested the effects of seven well-characterized mAbs against CHIKV E2 on ILE formation in human U-2 OS cells (Fig. 3). The approximate mAb binding sites are diagrammed in Fig. 3A and B, with details of the assays used to define the binding sites and functional effects of the mAbs summarized in Table S1. In brief, mAb chCHK-152 binds to residues on E2 domains A and B within a single E2 monomer (48–51). mAb C9^pMAZ^ binds across two E2 monomers within and/or between a trimer, interacting with residues on both E2 domains A and B (16, 50, 52, 53). mAbs ch-m242, K9-1, and D3-62 bind residues within E2 domain A (49, 54, 55) (T. Couderc and M. Lecuit, unpublished data). mAbs chCHK-265 and DC2.M108 bind residues within E2 domain B (48, 56–58). We confirmed by ELISA that all the mAbs bound to the surface of human U-2 OS cells infected with CHIKV 181/25 GFP (Fig. S1). Based on their half-maximal binding (EC_50_) values, the mAbs clustered into two groups: strong binders, including DC2.M108 (0.004 µg/mL), chCHK-152 (0.004 µg/mL), D3-62 (0.004 µg/mL), and K9-1 (0.011 µg/mL) and moderate binders C9^pMAZ^ (0.631 µg/mL), ch-m242 (1.62 µg/mL), and chCHK-265 (2.45 µg/mL). To test the effect of the Abs on ILE formation, we infected U-2 OS cells with CHIKV 181/25 GFP (MOI_U-2 OS_ 0.25) and then cultured the cells in the presence of 20 µg/mL of the indicated Ab starting at 2 hpi. This standard mAb concentration represented an 8- to >4,000-fold excess vs the determined EC_50_ for cell surface binding. Infection was allowed to progress in the presence of Ab for 9 h and ILEs were quantitated at 11 hpi by confocal microscopy (Fig. 3C and D’). Neither the A/B domain targeting mAbs chCHK-152 (1.6 ± 0.4, mean ± S.D.) and C9^pMAZ^ (1.1 ± 0.0) nor the B domain-specific mAbs chCHK-265 (1.0 ± 0.2) and DC2.M108 (1.5 ± 0.4) significantly reduced ILE formation compared to the no Ab control (1.0 ± 0.1) (Fig. 3C). However, all three domain A binding mAbs significantly reduced the number of ILEs formed per infected cell: ch-m242 moderately (0.8 ± 0.1) and K9-1 and D3-62 strongly (0.3 ± 0.1 and 0.2 ± 0.1). These data, thus, suggest an important role for E2 domain A in ILE formation.

### ILEs do not form in the absence of E1 expression

**Fig 4:**
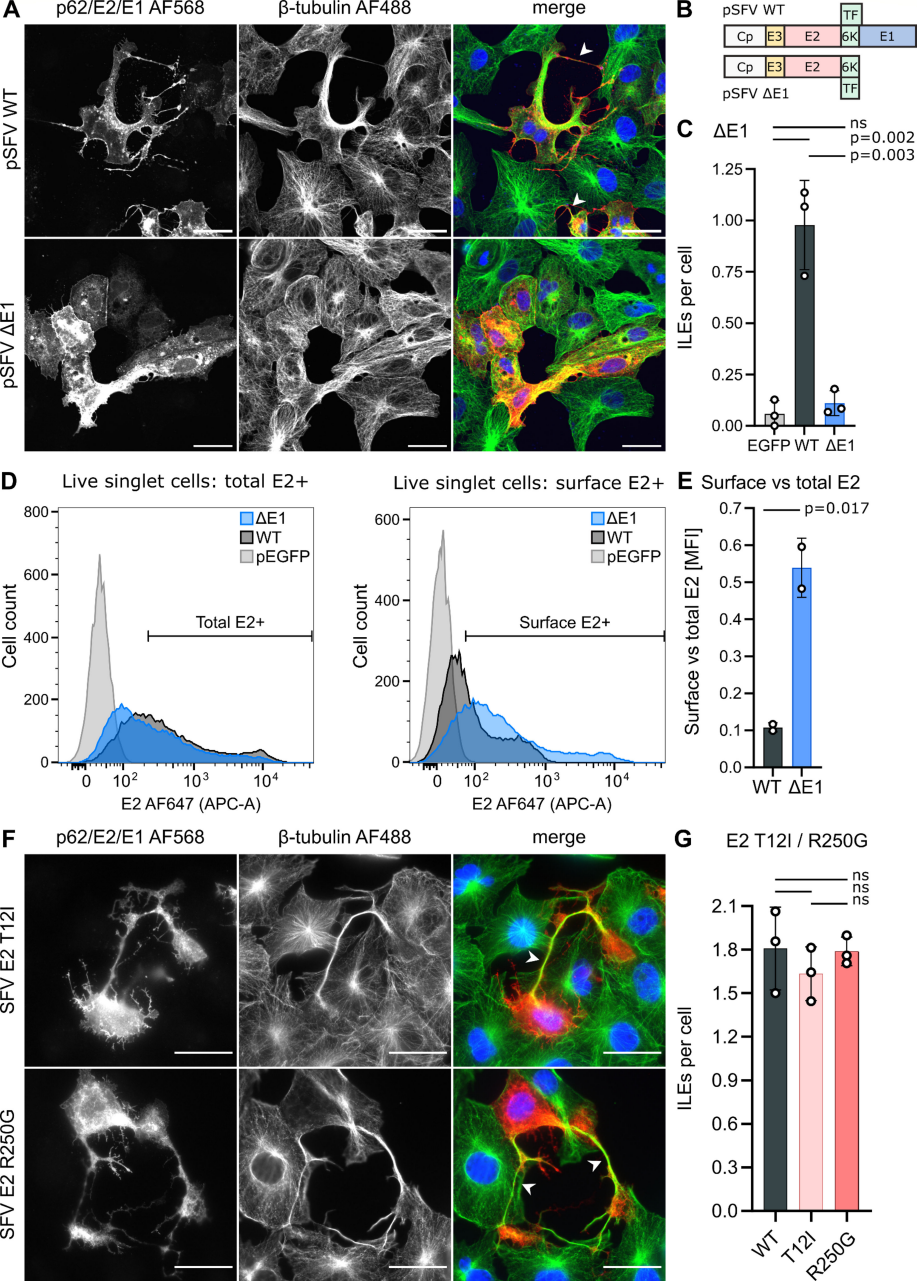
ILE formation requires E1 expression but is not affected by E2-E1 dimer stability. (A–C) Role of E1 in ILE formation. Vero cells were transfected with plasmids expressing the SFV structural proteins either with (pSFV WT) or without E1 (pSFV ΔE1). Samples were fixed at 24 hpt and ILEs were quantitated as described in Fig. 1. (D and E) Vero cells were transfected with pSFV WT, pSFV ΔE1, or an EGFP-expressing control vector pEGFP. At 24 hpt, cells were harvested and processed for analysis by flow cytometry: each condition was split into a non-permeabilized sample for E2 cell surface staining, and a matched permeabilized sample for total E2 staining. The histograms in panel D show the E2 fluorescent signal intensity profiles for the live singlet cell population. The bar graph in panel E represents the ratio of E2 surface signal vs E2 total signal, based on the mean fluorescence intensity of the respective E2+ cell populations. (F and G) Role of the E2-E1 dimer in ILE formation. Vero cells were infected with SFV WT, SFV E2 T12I, or SFV E2 R250G (MOI_Vero_ 0.30). Cells were fixed at 8 hpi cells, and ILEs were quantitated as described in Fig. 1. (F) For each condition, a representative single slice micrograph is shown; arrow heads point out ILEs; scale bar = 30 µm. (C, E, G) Bar graphs show the means of at least two biological replicates (white circles) ±S.D. Significance was determined as in Fig. 1. Total number of analyzed cells per condition: (C) pEGFP (124), pSFV WT (226), pSFV ΔE1 (326); (G) SFV WT (123), SFV E2 T12I (94), SFV E2 R250G (102).

In infected cells, p62/E2 forms a stable heterodimer with the viral fusion protein E1. While our data support a crucial role for p62/E2 (and their association with Cp) in the formation of ILEs, the importance of E1 was unknown. We, therefore, tested if ILEs could develop without the expression of E1. As E1 is essential for virus fusion, an E1 deletion virus is not infectious. To bypass this, we transfected Vero cells with expression constructs for either the WT structural proteins (pSFV WT) or a construct lacking the E1 coding region (pSFV ΔE1) (Fig. 4B). ILE formation was quantitated at 24 hpt (Fig. 4A and C). Loss of E1 expression blocked the formation of ILEs (0.1 ± 0.1 ILE/cell for pSFV ΔE1 transfected cells vs 1.0 ± 0.2 for pSFV WT expression). This defect in ILE formation is not due to inhibition of virus fusion, as fusion-deficient mutants of SFV (32) and CHIKV (Fig. S2) induce ILEs at levels similar to WT. Note that while ILE formation itself is independent of virus fusion, infection of a target cell through ILE-mediated cell-to-cell transmission requires virus endocytic uptake and low pH-triggered fusion (33).

Previous studies showed that E1 is required for virus budding and certain post-translational modifications of E2, but not for correct p62/E2 folding, cleavage and transport to the plasma membrane (59–63). We used flow cytometry to directly quantitate the levels of E2 on the plasma membrane in the presence or absence of E1 expression (Fig. 4D and E). WT or ΔE1-transfected cells were split into a permeabilized and a matched, non-permeabilized sample to detect the total or the cell surface levels of E2, respectively. The mAb E2-1 (64) used for detection recognized E2 in the absence of E1 expression (Fig. 4D), suggesting that E2 folds into an antigenically relevant conformation independent of E1 expression. Loss of E1 did not strongly reduce overall E2 expression (Fig. 4D, left) but increased the levels of E2 on the cell surface (Fig. 4D, right). The expression of the viral structural proteins alone results in the budding of virus-like particles (VLPs) from the plasma membrane (55), thereby removing envelope proteins from the cell surface. The defect in VLP budding in the ΔE1-transfected cells (61), thus, presumably results in the observed build-up of E2. In conclusion, ILEs were not formed even in the presence of an increased plasma membrane level of E2, suggesting that E1 is either directly required for ILE formation or that it supports important aspects of the E2 structure and/or post-translational modifications that promote ILEs.

### Perturbation of E2-E1 dimer stability does not attenuate the formation of ILEs

To more directly address the contribution of E1, we next tested if changes in E2-E1 heterodimer stability compromised ILE formation. We infected Vero cells with two well-characterized SFV mutants: E2 T12I which stabilizes the E2-E1 dimer and E2 R250G which destabilizes the dimer (65, 66). Analysis of ILEs at 8 hpi showed that neither mutant induced significantly different numbers of ILEs per cell than the SFV WT control (Fig. 4F and G). This result indicates that neither E2-E1 dimer stability nor the E2 T12 or R250 residues play a central role in ILE formation.

### Ab interaction with specific E1 domains attenuates ILE formation

**Fig 5:**
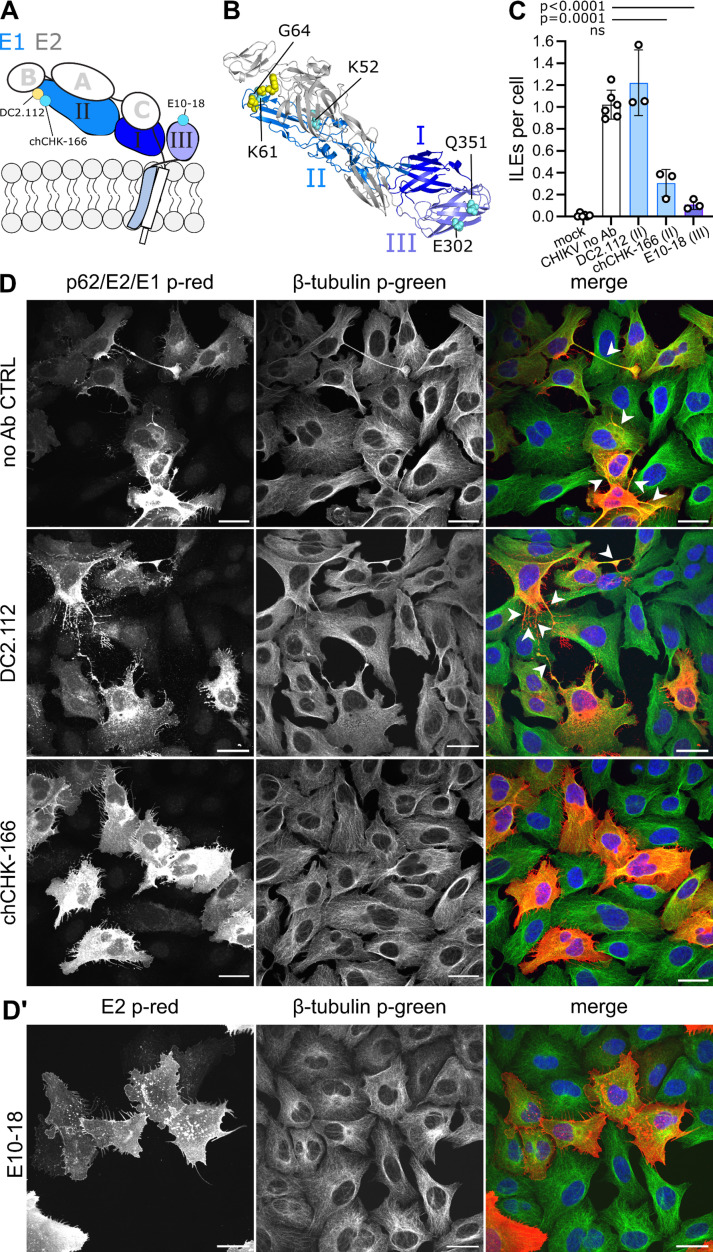
Masking of specific E1 domains by mAb binding attenuates ILE formation. (A) Schematic representation of the E2-E1 dimer, showing E1 and its domains I, II, III in blue and E2 and its domains A, B, C in gray. Highlighted are the approximate binding sites of the CHIKV E1 mAbs (as determined by neutralization escape mutants, alanine scanning, or structural studies, Table S1). (B) Crystal structure of the CHIKV E2-E1 dimer (PDB accession number 3J2W [49]) colored as in panel A. Critical epitope-binding residues (spheres) are shown in cyan for ILE-attenuating mAbs and in yellow for mAbs that did not reduce ILE formation. (C and D′) Effect of anti-E1 mAbs on ILE formation. Methods and analysis as described for the E2 mAbs in Fig. 3. For each condition, a representative single slice micrograph is shown; arrow heads point out ILEs; scale bar = 30 µm. (C) Bar graph shows the means of at least three biological replicates (white circles) ± S.D. Please note, subsets of mock and no Ab data were reproduced in Figure 3C, Figure 8B, and Figure S5 as respective experiments were done in parallel. Significance was determined as in Fig. 1. Total number of analyzed cells per condition: (C) mock (503), no Ab (507), DC2.112 (246), chCHK-166 (374), E10-18 (318).

Complete deletion of E1 perturbs multiple aspects of infection and, thus, did not allow us to conclusively define the role of E1 in ILE formation (Fig. 4). Our prior results showed that two mAbs against the CHIKV E1 protein decrease ILE formation in MEFs (and thus, decrease cell-to-cell transmission) (33). Here, we infected human U-2 OS cells with CHIKV and tested ILE formation in the presence of domain-specific anti-E1 antibodies. The E1 ectodomain contains three domains: the central domain I (DI), the elongated DII with the fusion loop at the tip, and the membrane proximal DIII which connects via a short stem to the helical TM domain (67, 68). The E1 ectodomain is oriented tangential to the virus membrane and associates with the E2 ectodomain in a “crossed fingers” like heterodimer arrangement (Fig. 3A and 4A) (47, 69). The fusion loop on DII is shielded by E2 domain B, and the E2 and E1 TM domains associate in the membrane (47, 69). To dissect the role of the E1 domains, we used three anti-CHIKV E1 mAbs: two that bind distinct residues in E1 DII, close to the fusion loop (chCHK-166 and DC2.112) (48, 51, 70), and one that binds to E1 DIII (T. Couderc and M. Lecuit, unpublished data) (Fig. 5A and B; Table S1). All three mAbs bound to the surface of human U-2 OS cells infected with CHIKV 181/25 GFP (Fig. S3) and were classified by their EC_50_ as strong binders DC2.112 (0.011 μg/mL) and chCHK-166 (0.005 µg/mL) or as moderate binders E10-18 (0.233 µg/mL). Similar to the tests of E2 mAbs, we infected human U-2 OS cells with CHIKV 181/25 GFP (MOI_U-2 OS_ 0.25), added 20 µg/mL mAb at 2 hpi and scored ILEs at 11 hpi by confocal microscopy (Fig. 5C and D). As previously observed in CHIKV-infected MEFs (33), mAbs E10-18 and chCHK-166 significantly reduced the number of ILEs formed per infected cell (0.3 ± 0.1 and 0.1 ± 0.0, respectively, vs the control 1.0 ± 0.1). Although mAb DC2.112 binds to a spatially similar epitope in E1 domain II as chCHK-166, it did not attenuate the development of ILEs (1.2 ± 0.3). Together, these data show that Ab interaction with specific regions in E1 domain DII or DIII can attenuate ILE formation.

### mAbs block ILE formation by masking specific spike regions

**Fig 6:**
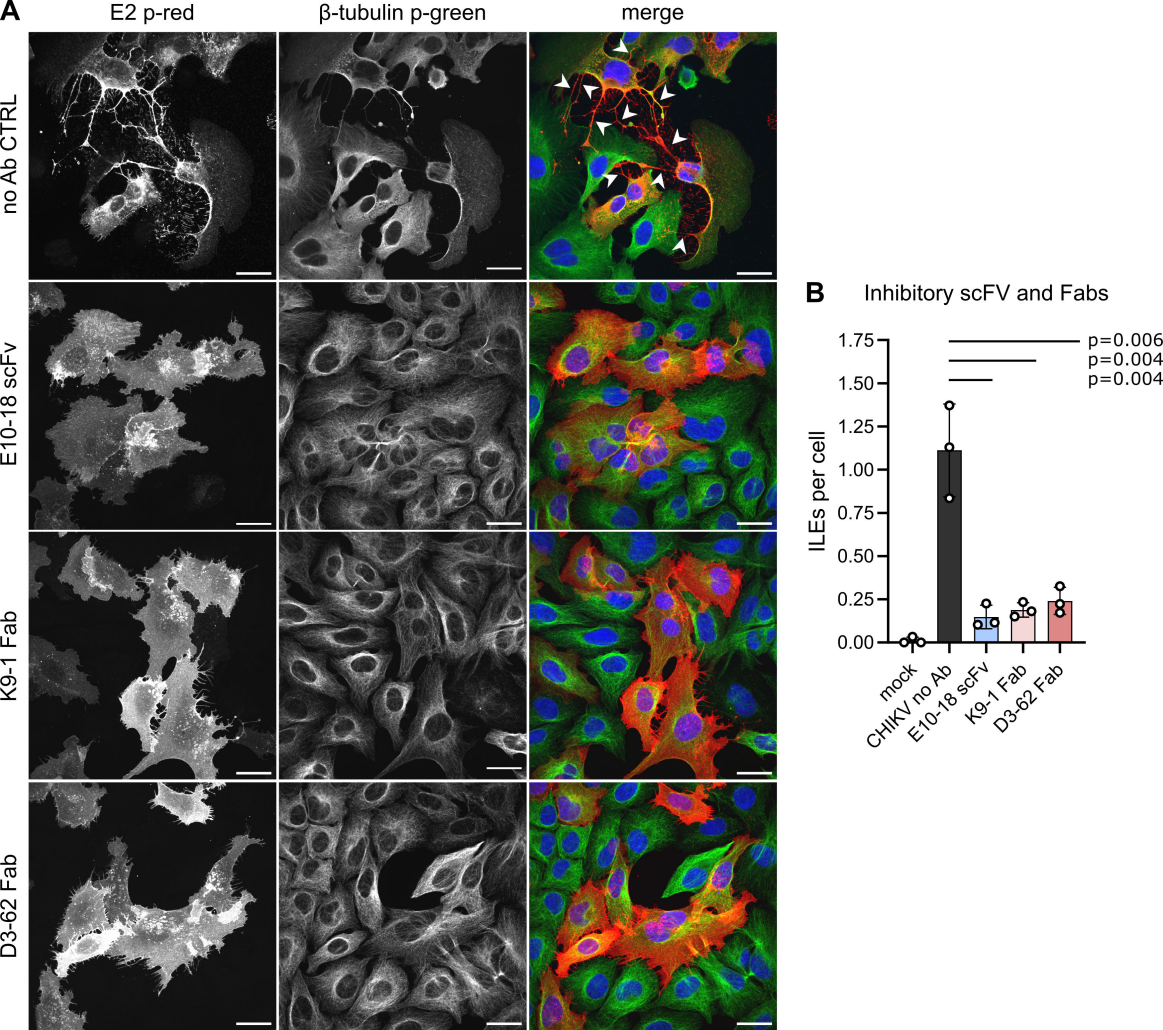
Monovalent fragments of anti-CHIKV mAbs E10-18, K9-1, and D3-62 attenuate ILE formation. U-2 OS cells were infected with CHIKV 181/25 GFP (MOI_U-2 OS_ 0.25) for 2 h, and then the inoculum was replaced with medium containing 20 µg/mL of the indicated Ab fragments: single-chain variable fragment (scFv) of mAb E10-18 and antigen-binding fragment (Fab) of mAbs K9-1 and D3-62. Method and analysis as described in Fig. 3. (A) For each condition, a representative single slice micrograph is shown; arrow heads point out ILEs; scale bar = 30 µm. For clarity, micrographs were pseudo-coloured to represent viral proteins in red and β-tubulin in green. (B) Bar graph shows the means of three biological replicates (white circles) ±S.D. Please note, subsets of mock and no Ab data were reproduced in Figure 3C as respective experiments were done in parallel.Significance was determined as in Fig. 1. Total number of analyzed cells per condition: mock (169), no Ab (197), E10-18 scFv (253), K9-1 Fab (323), D3-62 Fab (311).

We have, thus, identified three anti-E2 mAbs (ch-m242, K9-1, and D-62) and two anti-E1 mAbs (chCHK-166 and E10-18) that reduced ILE formation (Fig. S6A and B). Successful formation of an ILE requires two essential but poorly understood steps: (i) development of the extension itself, either through active outgrowth or by movement of the infected cell away from the target cell (32) and (ii) stable attachment of the extension tip to the target cell. While the underlying mechanism(s) for these steps are unclear, our results suggest that specific E1 and E2 epitopes need to be accessible at the cell surface for ILEs to form. We reasoned that the inhibitory mAbs could function either by sterically hindering access to essential spike regions or by reducing the number of E2-E1 proteins on the cell surface. Such downregulation of cell surface proteins has been observed for Abs that crosslink their target protein and trigger its endocytic clearance from the cell surface (71–73). Monovalent Ab fragments such as antigen-binding fragments (Fab) or single-chain variable fragments (scFv) that cannot crosslink do not induce endocytic uptake and degradation of their target proteins. Two of the ILE-attenuating mAbs, K9-1 and D3-62, caused a change of viral E2 staining compared to the no Ab control (Fig. 3D no Ab vs Fig. 3D’). As this patchy appearance might represent mAb-mediated crosslinking of E2, we tested if Fabs of the K9-1 and D3-62 mAbs or a scFv of the E10-18 mAb retained the ability to inhibit ILE formation in CHIKV-infected U-2 OS cells (Fig. 6A and B). Both the Fabs and the scFv significantly reduced the number of ILEs formed per infected cell compared to the no Ab control (Fig. 6A and B). This result suggests that epitope masking is the main mechanism of Ab-mediated inhibition of ILE formation.

### The ability to form ILEs is conserved across the four major CHIKV lineages

Many viruses, including CHIKV, evolved mechanisms to evade host immune responses to replicate and spread successfully within their (human) host (74, 75). In cell culture, ILEs allow CHIKV to spread efficiently from cell to cell in the presence of neutralizing Abs (33), suggesting that ILEs might contribute to CHIKV virulence in human patients.

**Fig 7:**
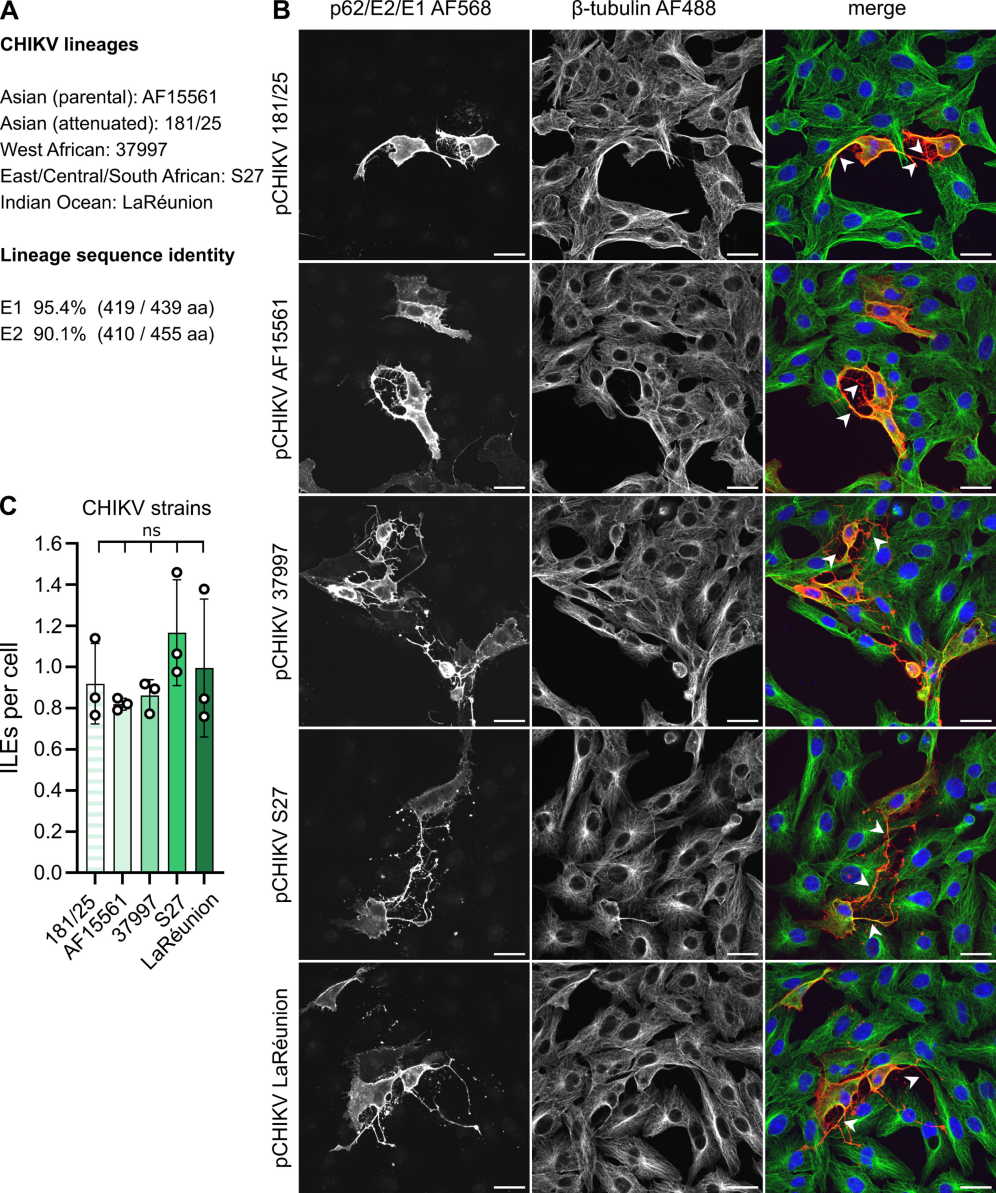
The ability to form ILEs is conserved across the four major CHIKV lineages. (A) The four major CHIKV lineages and respective CHIKV strains. The percent E2 and E1 sequence identity between the five strains is listed, and the number of identical amino acids vs total amino acids is shown in brackets. Sequences were aligned with Clustal Omega (78). (B, C) Vero cells were transfected with plasmids expressing the structural proteins of the indicated CHIKV strains. At 24 hpt, samples were fixed and analyzed as described in Fig. 1. (B) For each condition, a representative single slice micrograph is shown; arrow heads point out ILEs; scale bar = 30 µm. (C) Quantitation of ILEs formed per expressing cell. Bar graph shows the means of three biological replicates (white circles) ±S.D. Significance was determined as in Fig. 1. Total number of analyzed cells per condition: pCHIKV 181/25 (179), pCHIKV AF15561 (166), pCHIKV 37997 (162), pCHIKV S27 (157), pCHIKV LaRéunion (130).

CHIKV is classified into four major lineages: West African (WA); East, Central, and South African (ECSA); Indian Ocean (IOL); and Asian. Our experiments up to this point focused on the attenuated Asian CHIKV strain 181/25. To address if the ability to induce ILEs was conserved across all four lineages, including medically relevant virulent strains, we compared ILE formation of 181/25 with its virulent, parental strain AF15661 [see also reference (33)], the ECSA strain S27, the IOL strain LaRéunion 2006, and the WA strain 37997 (Fig. 7). Due to biosafety considerations, we induced ILE formation by expressing only the respective CHIKV structural proteins in Vero cells, in the absence of infection (Fig. 7B and C). All tested strains induced comparable numbers of ILEs per cell, independent of virulence and lineage. The AF15661 and 181/25 strains (both Asian lineage) differ in five amino acids, with two E2 substitutions, G82R and T12I being responsible for attenuation (76). Our data on dimer stability ruled out an effect of E2 T12I on ILE formation (Fig. 4G). The change from the parental E2 glycine 82 to arginine maps within E2 domain A, but our results (Fig. 7) indicate that while this substitution affects virulence (77), it does not affect ILE formation. Together, our data suggest that the capability to induce ILEs is a conserved feature of CHIKV infection across all four CHIKV lineages and might present a medically relevant target for anti-viral intervention.

In mice, efficient clearance of CHIKV from the circulation requires interaction of the cellular scavenger receptor MARCO with the conserved E2 domain B residue K200. Mutation of E2 K200 abrogates interaction with MARCO and, thus, prevents CHIKV clearance, causing increased viremia (79, 80). We tested whether the MARCO-mediated clearance-resistant CHIKV 181/25 E2 K200A mutant (81) might induce increased numbers of ILEs. Comparison of CHIKV 181/25 WT vs E2 K200A showed no significant difference in ILE formation (Fig. S4). This is in keeping with our finding that mAbs binding E2 domain B had no effect on ILE formation (Fig. 3).

### Serum or plasma from convalescent CHIKV patients decreases ILE formation

**Fig 8:**
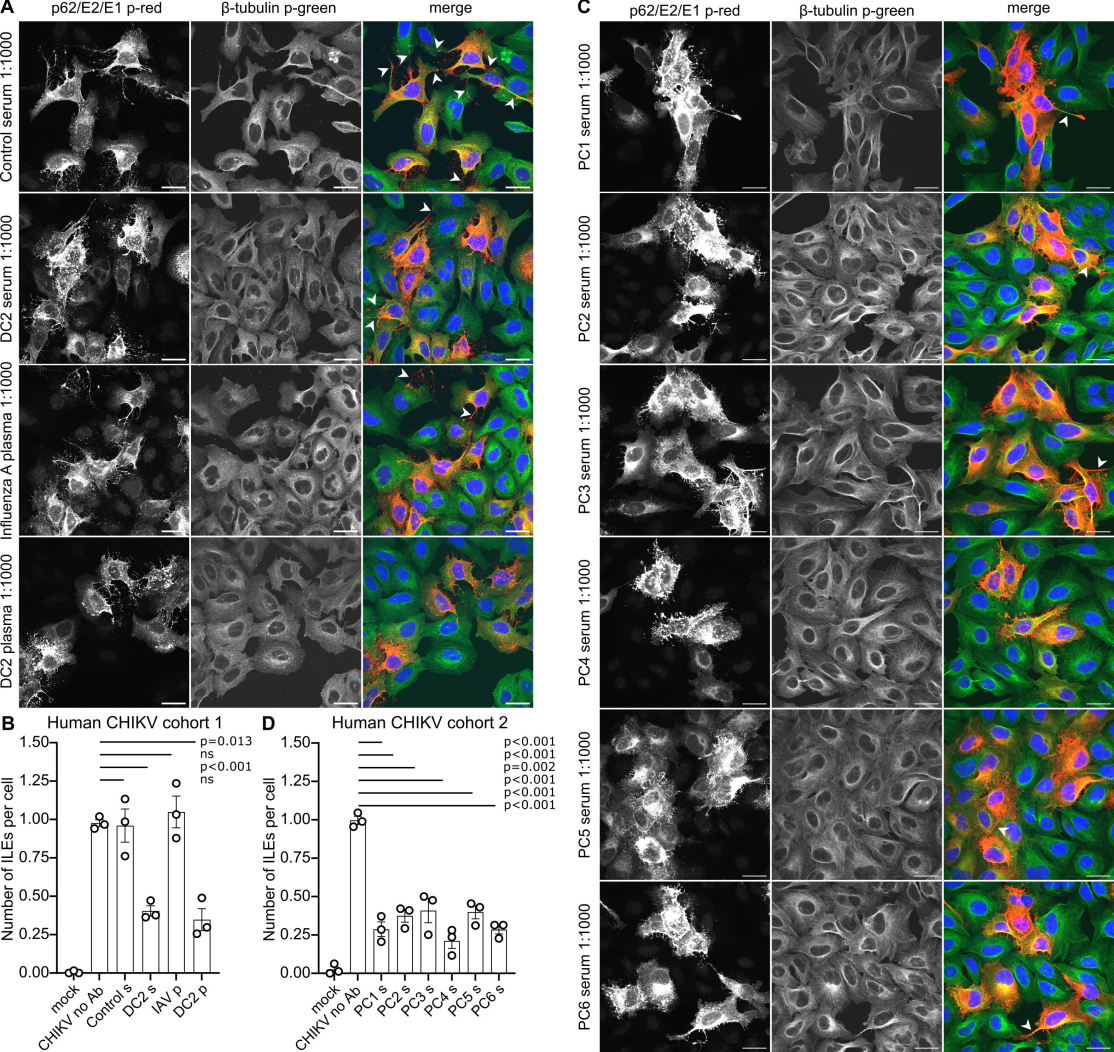
Human CHIKV patients can produce Abs that attenuate ILE formation. U-2 OS cells infected with CHIKV 181/25 GFP (MOI_U-2 OS_ 0.25) were incubated with growth medium (no Ab), or growth medium containing a 1:1,000 dilution of serum (“s”) or plasma (“p”) from either convalescent human CHIKV patients, a recent Influenza A (IAV) vaccinee, or a control volunteer who was seronegative for both CHIKV and IAV. Patient cohort 1 includes serum and plasma from a female patient infected with CHIKV in the Dominican Republic (DC2); patient cohort 2 includes sera from two female and four male patients infected with CHIKV in Puerto Rico (PC1-6). ILE formation was quantitated at 11 hpi as described in Fig. 1. (A, C) For each condition, a representative single slice micrograph is shown; arrow heads point out ILEs; scale bar = 30 µm. For clarity, micrographs were pseudo-coloured to represent viral proteins in red and β-tubulin in green. (B, D) Bar graphs show the means of three biological replicates (white circles) ±S.D. Please note for panel B that subsets of mock and no Ab data were reproduced in Figure 3C, Figure 5C, and Figure S5 as respective experiments were done in parallel. Significance was determined as in Fig. 1. Total number of analyzed cells per condition: mock (c1: 230, c2: 195), no Ab (c1: 234, c2: 222), control serum (237), DC2 serum (339), IAV plasma (225), DC2 plasma (291), PC1 serum (397), PC2 serum (326), PC3 serum (317), PC4 serum (344), PC5 (316), PC6 (330).

So far, ILEs have only been directly observed in cell cultures. However, data from mice infected with CHIKV suggest that ILEs also form *in vivo* and that they contribute to Ab-resistant virus spread in mice (33). In human patients, the acute phase of CHIKV infection is characterized by high fever, rash, and severe joint and muscle pain (reviewed in reference 82). Within days of infection, patients develop a robust Ab response that is essential for virus clearance and for protection from symptomatic re-infection in the future (83). Since specific purified anti-CHIKV mAbs reduced ILE formation, we tested if plasma or sera from convalescent human CHIKV patients could similarly inhibit ILE formation. CHIKV 181/25-infected U-2 OS cells were incubated with a 1:1,000 (Fig. 8) or a 1:100 (Fig. S5) dilution of heat-inactivated serum (“s”) or plasma (“p”) from convalescent human CHIKV patients. Patient cohort 1 includes serum and plasma from a female patient infected with CHIKV in the Dominican Republic (*D*ominican Republic, *C*HIKV: DC2) (51); patient cohort 2 includes sera from two female and four male patients infected with CHIKV in Puerto Rico (*P*uerto Rico, *C*HIKV; PC1-6). The CHIKV patient samples were compared to a no Ab control (no Ab), human control serum (seronegative for influenza A and CHIKV), and plasma from an influenza A virus (IAV) vaccine. The results showed that serum or plasma from all the CHIKV patients significantly reduced ILE formation, independent of age or sex (Fig. 8B and D). The inhibitory effect was specific to plasma/serum from CHIKV patients, with none of the control samples causing a significant reduction. Thus, human Abs induced during natural CHIKV infection can inhibit ILE formation in cell culture.

## DISCUSSION

Alphavirus infection of cultured cells can induce the formation of long filopodia-like extensions that mediate intercellular transmission, shielding virus from neutralization by extracellular Abs (32, 33). Our recent findings in a mouse model also support a contribution of ILEs and cell-to-cell transmission to CHIKV infection *in vivo* (33). However, despite emerging appreciation of the role of ILEs in CHIKV infection, our understanding of their formation and viral protein requirements is limited. Previous work demonstrated that the expression of the viral structural proteins alone induces ILE formation and that Cp and its interaction with E2 are required (32). Here, we defined the contribution of the E2, 6K/TF, and E1 proteins to ILE formation in cultured cells using a set of well-characterized SFV mutants and anti-CHIKV mAbs. The structural proteins 6K and TF were dispensable for ILE formation. In contrast, our data show that both E2 and E1 are essential for the formation of ILEs and highlight a central role for E2 domain A. Importantly, we also demonstrated that ILE formation can be attenuated not only by purified anti-CHIKV mAbs but also by sera from convalescent human CHIKV patients.

As there is no membrane continuity between the ILE and the target cell (32), the contact site must be tightly sealed to mediate the observed shielding of virions from high concentrations of neutralizing Abs (33). This suggests the presence of “ligand/receptor” pairs that produce very close apposition of the two cellular membranes, comparable to a tight or adherens junction. ILEs originate only from infected/viral structural protein-expressing cells but can attach to either infected/expressing or naïve cells (32). Our data suggest that the ligands on the ILE are comprised of E2-E1, and the block in ILE formation when critical epitopes are masked by mAbs (Fig. S6A and B) argues that the alphavirus structural proteins do not simply provide signals that promote cytoskeletal remodeling, but rather, directly mediate the cell-cell interactions that promote stable ILEs.

Although they are required, the viral spike proteins alone are not sufficient for ILE formation: our current and prior data show that Cp and its interaction with E2 are also essential, independent of their functions in virus budding (32). This was further supported here by several observations: although deletion of 6K/TF causes a strong budding defect (43), it did not attenuate ILE formation. The C9 ^pMAZ^ mAb prevents virus budding by bivalent bridging of adjacent spikes, thus increasing the distance between spikes and blocking membrane curvature (16, 50, 52, 53). This mAb did not attenuate ILE formation. As neither budding nor plasma membrane curvature appears essential for ILE formation, the precise mechanistic contribution/s of the Cp-E2 interaction to ILE formation remain to be determined. Based on our current findings, we hypothesize that Cp acts to cluster or organize the E2-E1 trimers in a specific orientation that renders the spikes “ILE-competent.”

The potential interacting partner/s on the target cell are also currently unknown, including whether they might interact directly with E2-E1. There does not appear to be a role for the CHIKV receptor MXRA8 or heparan sulfate proteoglycans on the target cell (32, 33), and the identity of the target cell partners remains an important question.

Our mAb data revealed a central role for E2 domain A in the formation of ILEs, while none of the tested E2 domain B mAbs affected ILE formation (Fig. S6A and B). ILE formation was most effectively disrupted by E2 domain A mAbs that bind epitopes near the E2 β-connector adjacent to domain B (i.e., mAbs K9-1, D3-62, ch-m242). As not all Abs to E2 domain A attenuated ILE formation, it is possible that only a specific region of E2 domain A is required or that the masking of domain A is affected by the angle of Ab binding or other Ab properties. Although the specific residues and regions remain to be defined, our data strongly support a role for E2 domain A in ILE formation, in keeping with the location of this domain at the top of the trimeric spike, where it would be readily accessible to interact with the target cell (Fig. S6A and B).

Our data showed that in addition to E2, E1 is also required for ILE formation. This is in agreement with the close associations of E2 and E1 in the heterodimer structure (47) and their frequent co-involvement in receptor interactions (84, 85). Similar to our studies with E2 mAbs, we tested several E1 mAbs for their effects on ILE formation. We confirmed here that mAb E10-18 potently attenuates the formation of ILEs (33) and showed that the scFv of this Ab also strongly inhibits. mAb E10-18 binds to E1 domain III and acts to alter the orientation of E2 domain A (F. Rey, T. Couderc, and M. Lecuit, unpublished data). Domain III is at least partially accessible in the spike trimer and is the site of VLDLR receptor binding in SFV (20). Thus, together the data suggest that mAb E10-18 inhibits ILE formation through its effects on the E2 protein. We also found that chCHK-166 potently attenuated ILE formation, in agreement with our prior results (33), while E1 mAb DC2.112 caused no inhibition. These two mAbs both bind near the fusion loop in E1 domain II and were reported to have partially overlapping epitopes (70). However, while both mAbs efficiently bind E1 on the surface of infected cells, only chCHK-166 efficiently binds and neutralizes virus particles (48), whereas DC2.112 particle recognition requires virus exposure to acidic pH (70). Since the fusion loop is relatively shielded by E2 in the neutral pH conformation of the spike, we speculate that the binding of chCHK-166 disrupts the interaction of the fusion loop with E2, while the binding of DC2.112 does not. The relative importance for ILE formation of chCHK-166 and E10-18 interactions with E1 vs their effects on the E2 heterodimer partner will be important to explore.

In this study, we tested a small number of anti-CHIKV mAbs (DC2.112, DC2.M108, C9^pMAZ^) that were originally isolated from human CHIKV patients and found that none inhibited ILE formation. As a broader approach, we tested sera from seven convalescent human CHIKV patients and found that all seven samples attenuated CHIKV-induced ILE formation in cell culture. This effect was specific as neither the IAV-immunized nor the uninfected control samples caused any significant inhibition of ILEs. This is the first evidence that during CHIKV infection humans can produce Abs that attenuate ILE formation in cell culture. The prevalence of such blocking Abs and the point at which they develop during disease remain to be determined.

In summary, our data support a working model in which trimeric E2-E1 spikes, organized by E2’s interaction with Cp, promote attachment between the tip of the ILE and its target cell, mediated by the interaction of E2 domain A with unknown partner proteins. Interesting questions are suggested by this model and by our results, including the mechanism by which E2-E1 mediates localized, spatially-confined attachment of ILE tips to the target cell, the mechanisms that maintain these stable cell-cell contacts, and the identity and function of potential interaction partners on the target cell. Addressing these questions will also help characterize the mechanisms that act to coordinate the stable ILE contacts and the dramatic remodeling of the cellular cytoskeleton within ILE, which are currently unknown. Further studies of alphavirus ILE will shed light on these mechanisms. They may also help define the functional importance of ILE in human alphavirus infections and the ability of antibodies and vaccines to target them.

## MATERIALS AND METHODS

### Human samples

Human samples from patient cohort 1 were provided by Dr. Jonathan Lai. Patient DC2 was identified as previously described (51); IAV plasma was obtained from a healthy IAV vaccinee; CHIKV/IAV double negative control serum was donated by a healthy individual; CHIKV immune status for samples was confirmed by ELISA. The human patient samples from cohort 2 were provided by Dr. William Messer. The six coded serum samples and associated demographic data were provided without sharing any protected health information as specified in MTA# 000316-2024. Convalescent CHIKV patients were identified in Puerto Rico either by PCR or IgM/IgG serology performed as part of a community serosurvey by the CDC in San Juan, Puerto Rico. Samples were collected between August and October 2023 as specified in the approved IRB protocol # 10212. All study participants were healthy at the time of blood draw; patients 2 and 6 are known to have contracted CHIKV in June and November of 2014, respectively. As a major CHIKV outbreak occurred in Puerto Rico between April and September 2014 (86), patients 1, 3, 4, and 5 are also assumed to have been infected in that time. Sex of human patients (where known): CHIKV/IAV double negative control serum (male, m); IAV control plasma (unknown); DC2 (female, f); PC1 (f); PC2 (m); PC3 (m); PC4 (m); PC5 (m); PC(6). Human serum and plasma samples were heat inactivated at 56°C for 30 min prior to use in any experiment.

### Cells

#### Cell lines for experiments

African Green Monkey Vero cells (ATCC; kind gift from Dr. Kartik Chandran, Einstein, Bronx, NY) were cultured in DMEM (Cytiva, Marlborough, MA) formulated with high glucose (4.5 g/L), L-Glutamine (4 mM), and sodium pyruvate (1 mM) and additionally supplemented with FBS (10%, vol/vol; GeminiBio, West Sacramento, CA), and Pen-Strep (100 units Penicillin/mL and 100 µg Streptomycin/mL; Gibco, Waltham, MA). Human osteosarcoma U-2 OS cells (ATCC, Manassas, VA) were cultured in McCoy’s 5A (modified) medium additionally supplemented with FBS (10%, vol/vol) and Pen-Strep.

#### Cell lines for virus production

Baby hamster kidney BHK-21 cells clone WI-2 were a gift from Dr. Ari Helenius, and BHK-21 clone C-13 were purchased from ATCC. Both BHK-21 cell lines were cultured in DMEM (Cytiva) formulated with high glucose (4.5 g/L), and L-Glutamine (4 mM), and additionally supplemented with FBS (5%, vol/vol: WI-2; 10%, vol/vol: C-13), Tryptose Phosphate Broth (10%, vol/vol; Gibco), and Pen-Strep.

Unless otherwise noted, all cells were grown and infected at 37°C and 5% CO_2_. Cell lines were routinely checked for Mycoplasma contamination using the MycoScope PCR Mycoplasma Detection Kit (Genlantis/Amsbio Cambridge, MA).

### Production of viral RNA and virus stocks

#### Alphavirus infectious clones

The wild-type pSP6-SFV4 infectious clone [SFV WT (87)] was used to generate the mutants SFV E2 T12I (65), SFV E2 R250G (66, 88), and SFV Cp MXI (35, 89). The CHIKV vaccine strain infectious clone pSinRep5-181/25ic [CHIKV 181/25 (77)] was a kind gift from Dr. Terence Dermody and was used to generate CHIKV 181/25 E1 F95A (90) and CHIKV 181/25 E2 K200A (79, 80). The GFP-expressing reporter virus CHIKV 181/25 GFP (91) was a kind gift from Dr. Elena Frolova.

Infectious clones were used to generate virus stocks as previously described (28, 45). In brief, infectious clone plasmids were *in vitro* transcribed using an SP6 RNA polymerase (Promega, Madison, WI). The resulting infectious viral RNA was either purified using the RNeasy MiniElute Kit (Qiagen, Germantown, MD) and stored at −80°C if used for forward transfection or was directly electroporated into BHK-21 cells for production of virus stocks. To obtain optimal virus titers, wild-type and mutant CHIKV stocks were produced in BHK-21/C-13 cells, and wild-type and mutant SFV stocks were produced in BHK-21/WI-2 cells (92). The cell medium was collected 24 h after electroporation and clarified by centrifugation (10,000 × *g*, 4°C, 10 min). The clarified supernatant was buffered with 10 mM HEPES pH 8.0, aliquoted, and frozen at −80°C. Frozen virus stocks were thawed and titered by focus-forming assay as described below.

### Virus titration by focus forming assay

Vero or U-2 OS cells were cultured in complete medium in 96-well plates for 20 h and then switched to 150 µL [MEM supplemented with 0.2%, wt/vol, bovine serum albumin (BSA), Pen-Strep, and 10 mM HEPES pH 7.0] and inoculated by addition of 50 µL of 10-fold serially diluted virus stocks. After incubation for 1 h (37°C and 5% CO_2_), the inoculum was replaced with 150 µL overlay made of a 1:1 mixture of [2% carboxymethylcellulose in Hanks salts solution] and [MEM plus heat inactivated FBS (4%, vol/vol), 2X Pen-Strep, L-Glutamine (4 mM; Gibco), and 20 mM HEPES pH 7.0]. Foci were allowed to develop at either 37°C (CHIKV), or 28°C (SFV) for 14–16 h. Samples were fixed with paraformaldehyde (PFA; 1%, vol/vol in PBS; Electron Microscopy Sciences, Hatfield, PA), washed extensively with 37°C pre-warmed PBS, and blocked with permeabilizing wash buffer [PBS plus BSA (0.1%, wt/vol) and saponin (0.1%, wt/vol)] at room temperature (RT) for 15 min. Foci were immuno-labeled for 2 h at RT with anti-E2 mAb (E2-1); clarified hybridoma supernatant (64) diluted 1:10 in permeabilizing wash buffer. Samples were washed with PBS, incubated at RT for 1 h with 50 µL HRP-conjugated secondary antibody [peroxidase-labeled anti-mouse IgG (H + L); Seracare No: 5450-0011, Milford, MA] diluted 1:1,000 in permeabilizing wash buffer, washed with PBS, and foci developed by incubation with 50 µL Kpl TrueBlue Peroxidase substrate (Seracare) at RT for 25 min. The peroxidase reaction was stopped by washing the plates extensively with MilliQ ultrapurified water. Plates were air-dried at RT and foci quantitated using an ImmunoSpot S6 Macroanalyzer with Biospot 7.0.9.10 software (Cellular Technologies, Shaker Heights, OH).

### Generation of pSFV and pCHIKV constructs

Plasmids expressing the SFV or CHIKV structural proteins (pSFV and pCHIKV) were generated by subcloning the respective viral ORF into the mammalian expression vector pcDNA3.1(−) (Invitrogen) by PCR. For the pSFV constructs, a Kozak sequence (5′-GCCACCATG-3′) was included and the following sequences were used as a template: pSP6-SFV4 (45), pSP6-SFV4/mutL (41), pSP6-SFV4/Y399R (18), or pSFV-SFV4/Δ6K/TF (45) using the forward primer: 5′-CATGGATCCGCCACCATGAATTACATCCC-3′, and the reverse primer: 5′-CATTAAGCTTTTATCTGCGGAGCCCA ATGC-3′. The pSFV ΔE1 expression vector was generated by subcloning the structural protein ORF from pSP6-SFV4, deleting the full E1 coding sequence, and adding a 3′ stop codon by PCR (reverse primer: 5′-ACTTAAGCTTTTAAGCTCTGGCGGTTGCCCCGAG-3′). For the pCHIKV expression constructs, a Kozak sequence was included (5′-NCCGCCACCATGG-3′) and the following CHIKV infectious clones were used as a template: CHIKV 181/25 (93) (kind gift from Dr. Terence Dermody, GenBank accession L37661.3); CHIKV 37997ic (94) (kind gift from Dr. Terence Dermody; GenBank accession AY726732.1); CHIKV AF15561 (77) (kind gift from Dr. Terence Dermody; GenBank accession EF452493.1); and CHIKV LaRéunion 2006 OPY-1 06-049 (95) (provided by Drs. Thérèse Couderc and Marco Vignuzzi; GenBank accession AM258994.1). The structural protein ORF for the pCHIKV S27 construct was inserted into the pcDNA3.1(−) backbone by GIBSON assembly of two human codon optimized fragments, using the S27 African prototype strain sequence (Uniprot accession Q8JUX5): the E3-E2-6K-E1 sequence was a kind gift from Dr. Kartik Chandran (51); the Cp coding sequence was synthesized by Integrated DNA Technologies (San Diego, CA). All pCHIKV constructs were validated for structural protein expression and VLP production by Western blot analysis. All sequences were verified by sequencing the complete ORF (Genewiz, South Plainfield, NJ).

### ILE formation

Vero or U-2 OS cells were seeded in µ-Slide 8 Well Glass Bottom imaging slides (Ibidi, Gräfelfing, Germany) in their respective cell medium and cultured for 24 h. For analysis by infection, cells were inoculated with SFV or CHIKV 181/25 GFP in the respective cell base medium supplemented with 0.2% BSA and 10 mM HEPES pH 7.0 for 1 h and then incubated in the respective cell complete medium for 8 hpi (SFV) or 11 hpi (CHIKV). Note on the choice of cell lines: experiments with infectious CHIKV were conducted in the human cell line U-2 OS as these cells are readily infected, form ILEs, and are optimal for confocal imaging. As SFV infection in U-2 OS does not induce ILE formation, experiments with infectious SFV were conducted in Vero (monkey) cells that are readily infected, imaged, and produced ILEs. Alternatively, Vero cells were forward transfected with 600 ng of the indicated CHIKV viral RNA and 0.6 µL lipofectamine 2000 (Invitrogen, Waltham, MA, USA) as per manufacturer’s instructions. In brief, lipofectamine and viral RNA were each diluted in separate 75 µL aliquots of Opti-MEM (Gibco), incubated at RT for 5 min, mixed, and incubated for an additional 30 min. The cells were washed once with RNAse-free PBS (Gibco) and incubated with the 150 µL transfection mix for 2 h. The medium was then exchanged for a complete growth medium and incubation continued until 11 hpi. For tests of protein expression, Vero or U-2 OS cells were forward transfected with 500 ng plasmid and 0.75 µL lipofectamine 2000 as above. After incubation with the 150 µL transfection mix for 5 h, the medium was exchanged for a complete growth medium and incubation continued for an additional 19 h. Following the incubation periods, all cell samples were fixed with PFA and processed for confocal microscopy as described below.

### Effect of Abs on ILE formation

U-2 OS cells seeded in µ-Slide 8 Well Glass Bottom imaging slides (Ibidi) were infected with CHIKV 181/25 GFP as described above. At 2 hpi, the medium was exchanged for 300 µL complete Vero growth medium containing either no Ab (no Ab control); or 20 µg/mL of the indicated mAb, Fab or scFv to CHIKV E1 or E2, or 1:100 or 1:1,000 dilutions of heat-inactivated human serum or plasma. Cells were incubated for an additional 9 h and fixed with PFA at 11 hpi and processed for microscopy as described below.

### Anti-CHIKV Abs and fragments

The following Abs are monoclonal, unless noted otherwise, and recognize the indicated CHIKV viral epitope (see also Table S1). E10-18 (anti-E1), K9-1 (anti-E2), and D3-62 (anti-E2) were purified from hybridoma supernatants from mice immunized with the p62-E1 heterodimer from the CHIKV La Réunion 2006 clinical isolate 05-115 as previously described for E10-18 (33), and kindly provided by Drs. Thérèse Couderc and Marc Lecuit. The corresponding K9-1 and D3-62 antigen-binding fragments (Fabs), and the E10-18 single-chain variable fragment (scFv) were expressed in stably transfected *Drosophila melanogaster* Schneider 2 cell lines and purified by affinity chromatography using StrepTactin columns and were provided by Dr. Félix Rey. chCHK-166 (anti-E1), ch-m242 (anti-E2), chCHK-265 (anti-E2), and chCHK-152 (anti-E2) were chimerized on human constant domains by transiently expressing the respective variable domain sequences and a human constant domain in FreeStyle 293F cells from pMAZ-IgL and pMAZ-IgH plasmids, followed by Protein A chromatography purification (Thermo Fisher Scientific, Wlatham, MA) as previously described (51, 96). Similarly, the C9^pMAZ^ Ab sequence (52), synthesized by Integrated DNA Technologies (San Diego, CA), was inserted into pMAZ-IgL and pMAZ-IgH-KRRG for recombinant expression on a human constant domain backbone (51). C9^pMAZ^ Ab was expressed in ExpiCHO-S cells (97) and purified by standard Protein A chromatography (Thermo Fisher Scientific, Wlatham, MA). The mAbs DC2.112 (anti-E1) and DC2.M108 (anti-E2) were previously isolated from a convalescent CHIKV patient (51, 57). The mAbs chCHK-116, ch-m242, chCHK-265, DC2.112, and DC2.M108 were provided by Dr. Jonathan Lai (48, 49, 51, 54–58, 70), and chCHK-152 was supplied by Dr. Zachary Bornholdt (Mapp Biopharmaceutical). The mAb DEN-4G2 to the flavivirus E protein was produced in hybridoma cells and purified from cell supernatant (98).

### Cell-surface ELISA

A cell-surface ELISA was used to quantify the binding of CHIKV mAbs to the surface of CHIKV-infected cells adapted from a previously described protocol (33). Briefly, U-2 OS cells in 96-well plates were infected with CHIKV 181/25 GFP (MOI 5) for 2 h at 37°C. At 11 hpi, cells were fixed with PFA without permeabilization and incubated with serial dilutions of mAbs (1  h, RT), and primary mAb binding was detected by HRP-conjugated goat anti-mouse (1:1,000, SeraCare, 5450-0011) or goat anti-human IgG (1:1,000, SeraCare, 5450-0009), using Ultra-TMB colorimetric substrate (Thermo Fisher #34028). The colorimetric reaction was quenched with 2N H_2_SO_4_ and absorbance at 450 nm was measured on a VictorX5 Multilabel plate reader (PerkinElmer, Shelton, CT).

### Immunofluorescent staining and microscopy

Samples were washed with 37°C PBS before fixation with 1% PFA in PBS at RT for 20 min. Unless noted otherwise, the following incubation steps were conducted in the dark. Cells were washed and blocked with permeabilizing blocking (PB) buffer (PBS containing 5% BSA and 0.2% Triton X-100) at RT for 3 × 5 min. Samples were incubated at 4°C overnight with primary Abs diluted in PB buffer: SFV- or CHIKV-infected cells were labeled with either polyclonal rabbit anti-p62/E2-E1 serum (1:800) or the mouse monoclonal anti-E2 Ab 20.2.9 (hybridoma supernatant, 1:50) (64) (Table 1). Microtubules were labeled with either a mouse or rabbit monoclonal anti-β-tubulin Ab (mouse: E7, 1:400; DSHB, Iowa City, IA, USA; rabbit: 9F3 #2128, 1:50; Cell Signaling Technology, Beverly, MA) (Table 1). The samples were washed 3 × 5 min at RT with PB buffer and were then incubated for 2.5 h at RT with secondary Abs and Hoechst 33342 (1:5,000, Invitrogen, Waltham, MA), diluted in PB buffer. Please refer to Table 1 for the combinations of primary and secondary Abs that were used. Samples were washed again 3 × 5 min at RT with PB buffer and imaged in PBS. All samples apart from Fig. 4F were imaged on a Nikon Spinning CSU-W1 Spinning Disc confocal microscope (Nikon, Melville, NY) with a 60× oil objective, acquiring *z*-stacks with Δ*z* = 0.3 µm and a minimal total *z*-volume of 3 µm. Due to instrument availability, samples in Fig. 4F were acquired on a DeltaVision Core Inverted Olympus IX71 microscope (Olympus, Waltham, MA), with a 60× oil objective, acquiring *z*-stacks with Δ*z* = 0.4 µm and a minimal total *z*-volume of 4.4 µm. Microscope settings such as laser power, exposure time, and scanning speed were kept constant between experiments.

**TABLE 1:** Primary and secondary Abs used in immunofluorescent microscopy^*e*^

Figure panel	Primary anti-VP Ab (detection)	Primary anti-VP (experimental)^*a*^	Primary anti-tub Ab	Secondary Ab (anti IgG H + L)^*c*^
Fig. 1B, E, 3A, F and 6A; Fig. S2A and S4A	Anti-p62/E2-E1 serum (p, rb)	n.a.	Anti-β-tub (m, ms)	VP: gt anti-rb AF568 (A11011) Tub: gt anti ms AF488 (A11001)
Fig. 7A and B; Fig. S5A	Anti-p62/E2-E1 serum (p, rb)	Anti-CHIKV serum (p, hu)	Anti-β-tub (m, ms)	VP: gt anti rb AF640 (A21244) Tub: gt anti ms AF568 (A11004)
Fig. 2D and 4D	Anti-p62/E2-E1 serum (p, rb)	chCHK152, chCHK265, ch-m242, chCHK166 (m, chimeric^*b*^) C9^pMAZ^, DC2.M108, DC2.112 (m, hu)	Anti-β-tub (m, ms)	VP: gt anti rb AF640 (A21244) Tub: gt anti ms AF568 (A11004)
Fig. 2D and 4D	Anti-E2 (IgG2a, m, ms)	K9-1, D3-62 IgG1 (anti-E2, mono, ms) E10-18 IgG2a (anti-E1, mono, ms)	Anti-β-tub (m, rb)	VP: dn anti ms AF640 (A31571)^*d*^ Tub: gt anti rb AF568 (A11011)
Fig. 5A	Anti-E2 (IgG2a, m, ms)	K9-1, D3-62 Fab (anti-E2, m, ms)^*d*^ E10-18 scFv (anti-E1, m, ms)	Anti-β-tub (m, rb)	VP: dn anti ms AF640 (A31571) Tub: gt anti rb AF568 (A11011)

^*a*^ Experimental Abs were used at 20 µg/mL, as described in “Effect of Abs on ILE formation,” above, and sources were as described in “Anti-CHIKV Abs and fragments,” above.

^*b*^ Chimeric Abs: mouse Fv on human constant domain backbone.

^*c*^ All secondary Abs are polyclonal and cross-adsorbed and were sourced from Invitrogen (Waltham, MA), catalog number in brackets.

^*d*^ Secondary Ab detects both the primary detection and experimental anti-VP Abs.

^*e*^ m = monoclonal, p = polyclonal, ms = mouse, rb = rabbit, gt = goat, dn = donkey, hu = human, VP = viral protein, tub = tubulin.

### ILE quantitation

All microscopy images were manually analyzed using the Fiji software (99), scoring an ILE if the following conditions were met (as defined by (32, 33): the extension (i) emanates from an infected cell, (ii) contacts a target cell, (iii) is tubulin positive, and (iv) is longer than 10 µm. Branched ILEs were counted as a single ILE; cells with extensions that contacted their own cell body (i.e., where the ILE producing cell was also the target cell) were excluded from analysis. To account for the 3-dimensional nature of ILEs, they were traced through different planes within a *z*-stack if required. Co-localization of tubulin and the extension was assayed in single slice images rather than in *z*-projections. Per condition, a minimum of 94, and a maximum of 374 individual, infected cells were analyzed (cumulative across three biological replicates).

### Surface and total E2 level analysis by flow cytometry

Vero cells in six well plates (Corning, Corning, NY) were forward transfected with pSFV WT or pSFV ΔE1 plasmid (5 µg) and lipofectamine 2000 (5 µL; Invitrogen) as per manufacturer’s instructions. Briefly, the lipofectamine and plasmid DNA were each diluted separately in 500 µL Opti-MEM (Gibco), incubated at RT for 5 min, mixed, and incubated for an additional 30 min. Cells were incubated with the 1 mL transfection mix for 5 h, and medium exchanged for complete Vero growth medium and incubation continued for an additional 19 h. Cells were then washed once with 37°C PBS and detached from the plate with Accutase at 37°C for 3 min (Millipore Sigma, Burlington, MA, USA). All subsequent steps were conducted either on ice, or at 4°C using ice-cold solutions and reagents. Unless otherwise noted, samples were washed with flow cytometry (FC) buffer (PBS containing 2% BSA and 15 mM HEPES pH 7.0). Samples were transferred to 1.5 mL tubes and were washed by centrifugation (600 rcf, 5 min, 4°C) first with Vero medium and then with FC buffer. Next, each specimen was split into a permeabilized and a non-permeabilized sample. The permeabilized sample was resuspended and fixed in PBS containing 2% PFA for 30 min, washed twice, and treated with FC buffer containing 0.2% TWEEN-20 for 20 min and washed again. SFV E2 was detected by staining with mAb E2-1, using the clarified hybridoma supernatant diluted 1:2 in FC buffer, 100 µL per sample, 1 h on rotator). Samples were washed three times and stained with anti-mouse AlexaFluor 647 secondary Ab (1:500 diluted in FC buffer, 100 µL per sample, 30 min on rotator; Invitrogen). The stained cells were then washed three times, fixed again as above, and washed once before being resuspended in 500 µL FC buffer. Non-permeabilized sample was stained in parallel, following the same protocol as for the permeabilized sample but omitting the first fixation and permeabilization step. All samples were analyzed using an LSR II cytometer (BD Biosciences, Franklin Lakes, NJ), acquiring 15,000 cells per condition. Samples were further analyzed using the FlowJo software version 10.2 (BD Biosciences), gating for (i) live cells using forward and side scatter, (ii) singlet cells, and (iii) E2 expressing cells based on AF647 signal intensity.

### Statistics

Sample statistics such as mean, standard deviation, and significance were calculated using the Prism software version 10.1.0 (GraphPad, Boston, MA). Signficance was determined by non-parametric, unpaired, two-tailed student *t*-test, using the means of at least three biological repeats. Differences were considered significant if *P* < 0.05. In the figures, *P* values were rounded to three decimal points. Unless noted otherwise, all data are represented as mean ± standard deviation.

## SUPPLEMENTAL MATERIAL

Figure S1 mbio.01986-24-s0001.tif Cell surface ELISA of anti-CHIKV E2 mAbs.

Figure S2 mbio.01986-24-s0002.tif ILE formation does not require virus fusion.

Figure S3 mbio.01986-24-s0003.tif Cell surface ELISA of anti-CHIKV E1 mAbs.

Figure S4 mbio.01986-24-s0004.tif CHIKV E2 K200 is dispensable for ILE formation.

Figure S5 mbio.01986-24-s0005.tif Human CHIKV patients can produce Abs that attenuate ILE formation.

Figure S6 mbio.01986-24-s0006.tif Critical binding residues of anti-CHIKV E1 and E2 mAbs in the context of the CHIKV E2/E1 trimeric spike.

Legends mbio.01986-24-s0007.docx Supplemental figure legends.

Table S1 mbio.01986-24-s0008.docx Characteristics of anti-CHIKV antibodies used in this study.

ASM does not own the copyrights to Supplemental Material that may be linked to, or accessed through, an article. The authors have granted ASM a non-exclusive, world-wide license to publish the Supplemental Material files. Please contact the corresponding author directly for reuse.
