# Supplementary figures and images for: The alphavirus determinants of intercellular long extension formation
Source: mBio. 2024 Dec 19;16(2):e01986-24. doi: 10.1128/mbio.01986-24 (PMC11796390; doi:10.1128/mbio.01986-24)

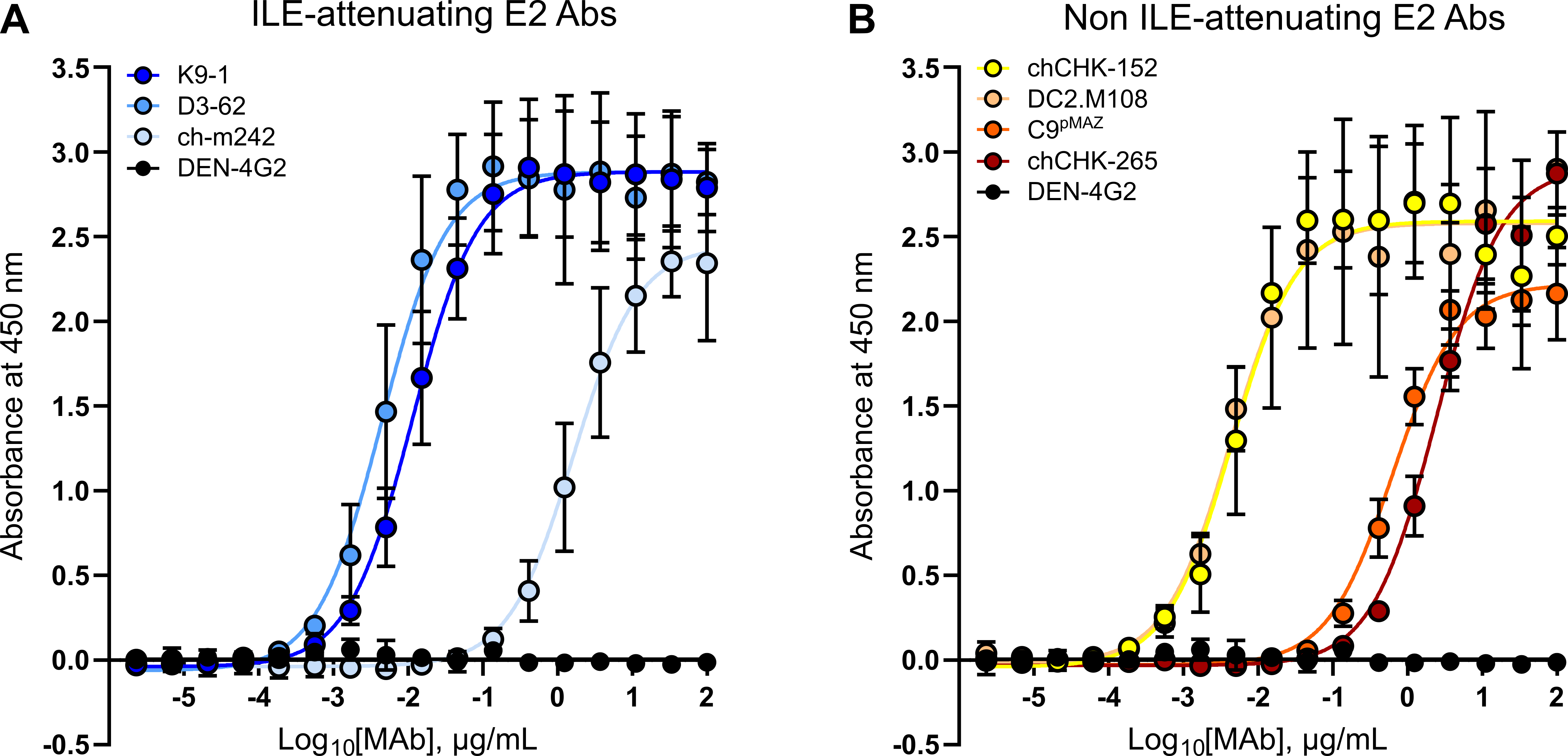

Supplement: Figure S1 — Cell surface ELISA of anti-CHIKV E2 mAbs. [file mbio.01986-24-s0001.tif]

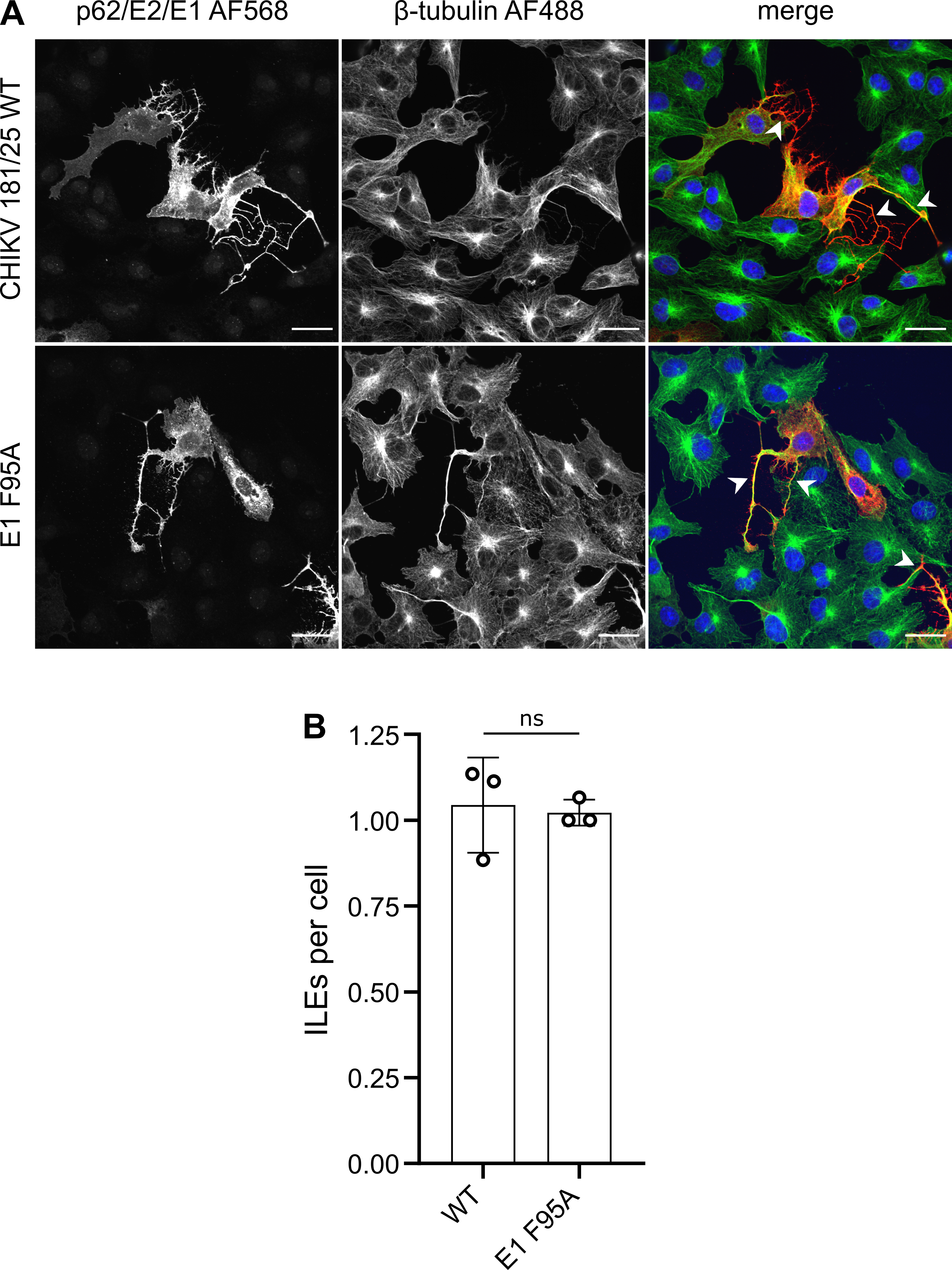

Supplement: Figure S2 — ILE formation does not require virus fusion. [file mbio.01986-24-s0002.tif]

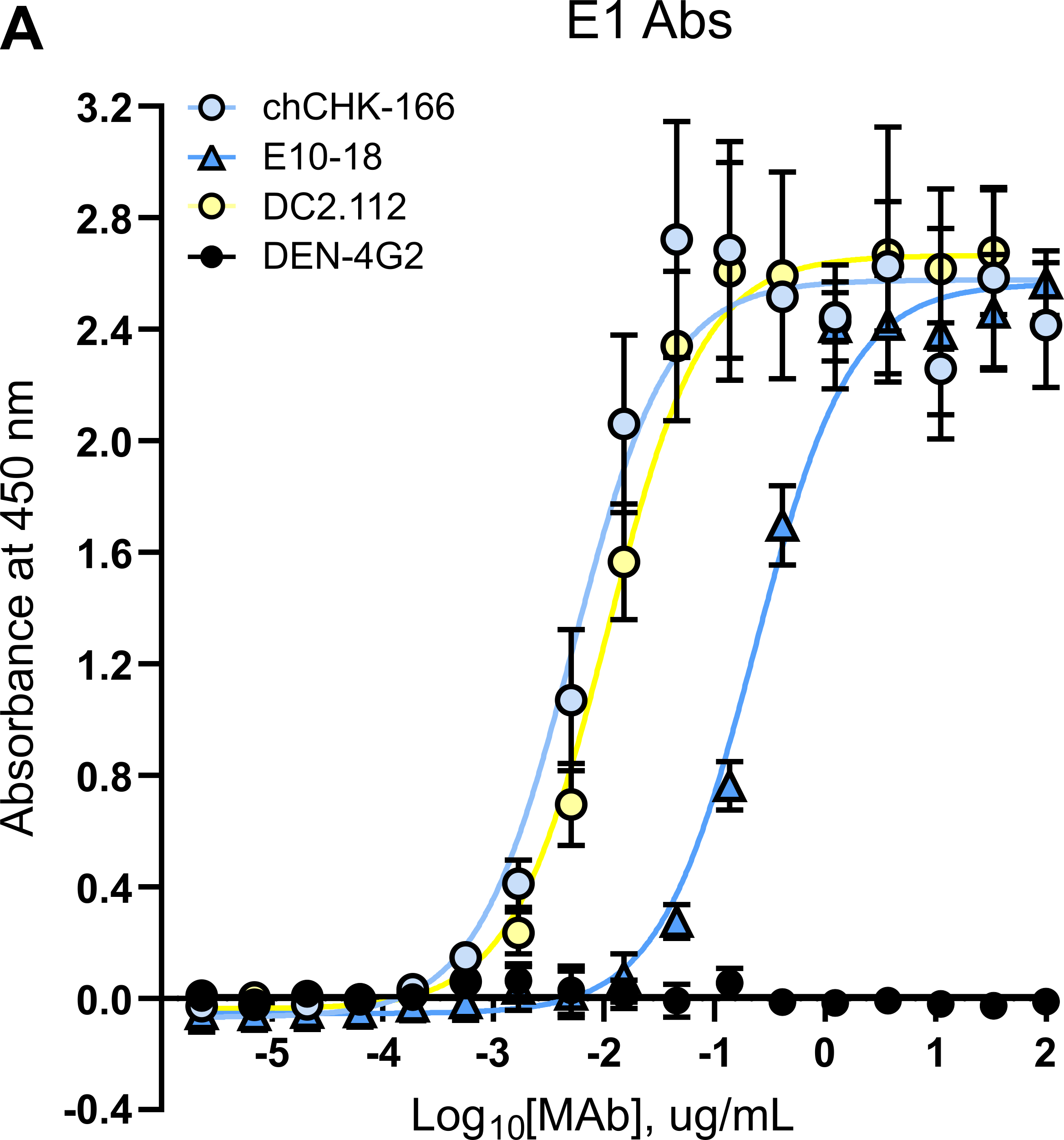

Supplement: Figure S3 — Cell surface ELISA of anti-CHIKV E1 mAbs. [file mbio.01986-24-s0003.tif]

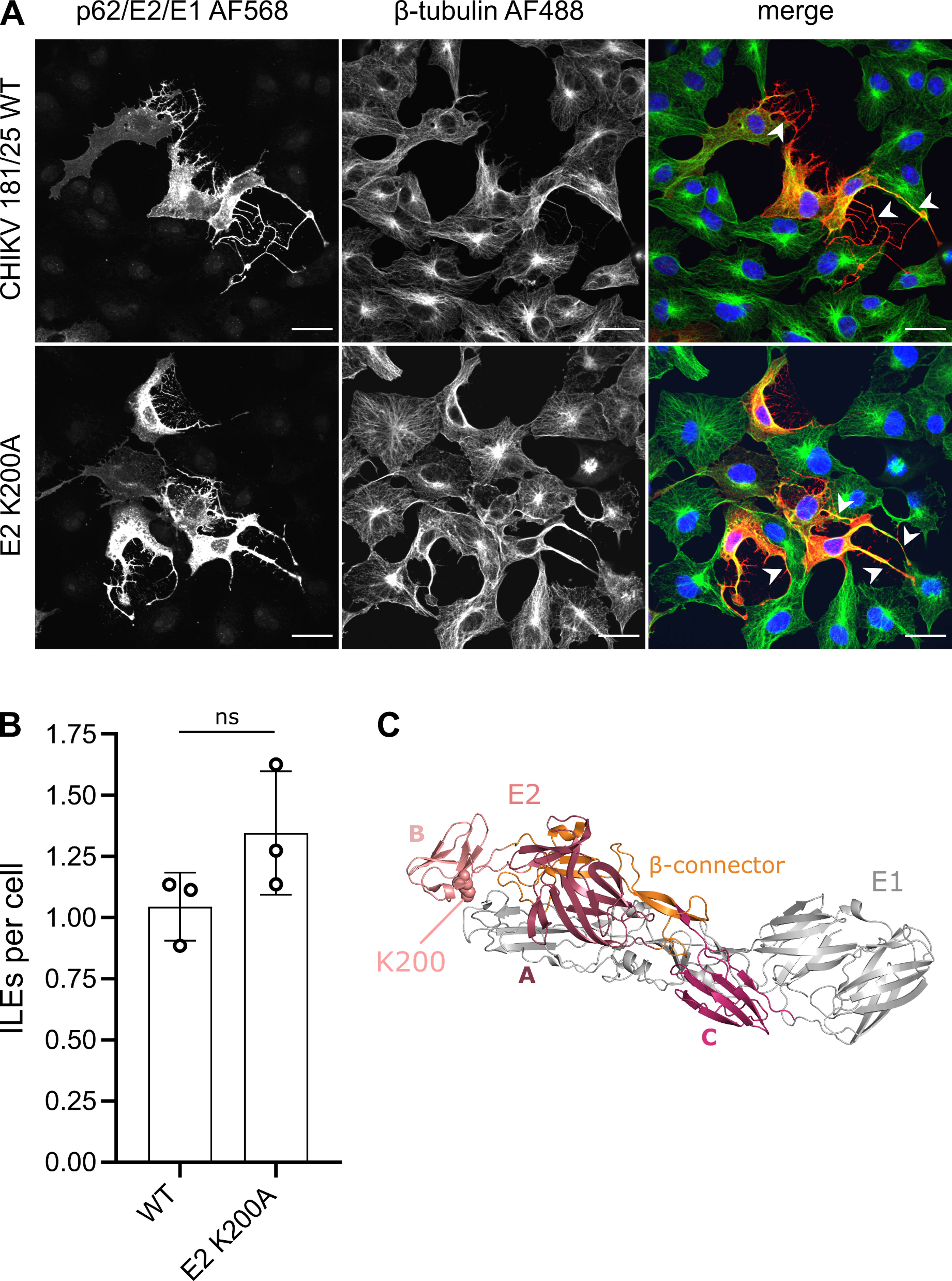

Supplement: Figure S4 — CHIKV E2 K200 is dispensable for ILE formation. [file mbio.01986-24-s0004.tif]

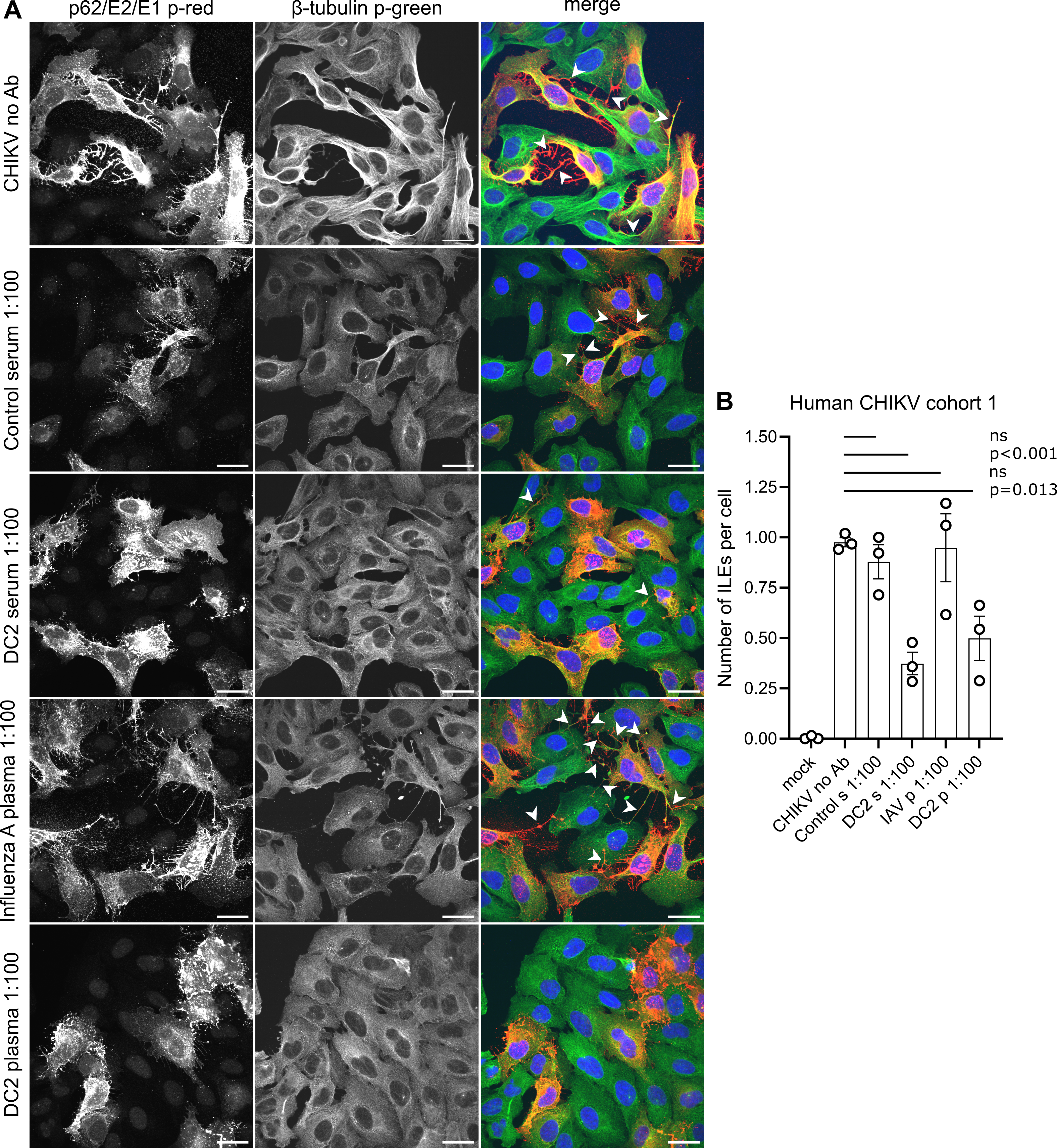

Supplement: Figure S5 — Human CHIKV patients can produce Abs that attenuate ILE formation. [file mbio.01986-24-s0005.tif]

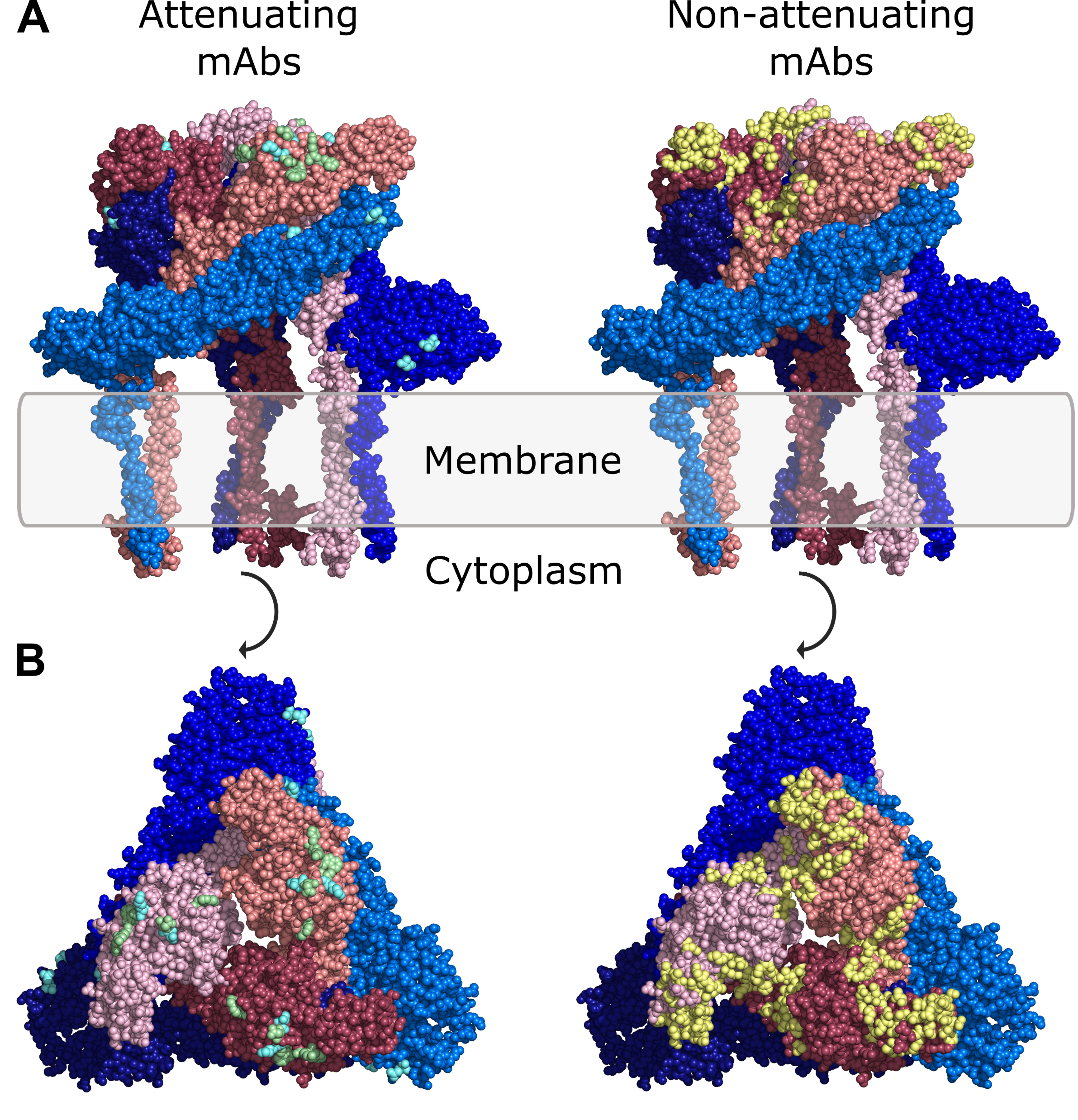

Supplement: Figure S6 — Critical binding residues of anti-CHIKV E1 and E2 mAbs in the context of the CHIKV E2/E1 trimeric spike. [file mbio.01986-24-s0006.tif]
