# Supplementary material for: The alphavirus determinants of intercellular long extension formation
Source: mBio. 2024 Dec 19;16(2):e01986-24. doi: 10.1128/mbio.01986-24 (PMC11796390; doi:10.1128/mbio.01986-24)
Supplement: Legends — Supplemental figure legends. [file mbio.01986-24-s0007.docx]

**SUPPLEMENTARY FIGURE LEGENDS**

**Figure S1: Cell surface ELISA of anti-CHIKV E2 mAbs.** Binding of anti-CHIKV E2 mAbs to the surface of CHIKV 181/25 GFP infected U-2 OS cells was confirmed by fixed cell ELISA as described in the methods. The mAbs that attenuated ILE formation are shown in shades of blue [A] and those that did not significantly reduce ILE formation in shades of yellow-red [B]. Lines represent nonlinear regression curves calculated based on Log (agonist) vs. response (three parameters). Please note that the negative control anti-dengue virus mAb (DEN-4G2), shown in black, is the same in [A] and [B] as experiments were done in parallel and split into two graphs for visibility.

**Figure S2: ILE formation does not require virus fusion.** [A-B] Vero cells were transfected with viral RNAs from either CHIKV 181/25 WT, or the fusion-negative CHIKV 181/25 E1 F95A mutant^89^. Samples were fixed at 11 hpi and ILEs were quantitated as described in Figure 1. [A] For each condition a representative single slice micrograph is shown; arrow heads point out ILEs; scale bar = 30μm. [B] Bar graph shows the means of 3 biological replicates (white circles) ± S.D. Significance was determined as in Fig. 1. Total number of analyzed cells per condition: CHIKV 181/25 WT (185), CHIKV 181/25 E1 F95A (220). Please note that the micrograph and data for CHIKV 181/25 WT were reproduced in Figure S4 as experiments were performed in parallel.

**Figure S3: Cell surface ELISA of anti-CHIKV E1 mAbs.** Binding of anti-CHIKV E1 mAbs to the surface of CHIKV 181/25 GFP infected U-2 OS cells was confirmed by fixed cell ELISA. The mAbs that attenuated ILE formation are shown in shades of blue and those that did not significantly reduce ILE formation in shades of yellow. Line represents nonlinear regression curve calculated based on Log (agonist) vs. response (three parameters). The negative control anti-dengue virus mAb (DEN-4G2) is shown in black and is the same as in Figure S1 since experiments were performed in parallel.

**Figure S4: CHIKV E2 K200 is dispensable for ILE formation.** [A-B] Vero cells were transfected with viral RNAs from either CHIKV 181/25 WT, or the CHIKV 181/25 E2 K200A mutant. Samples were fixed at 11 hpi and ILEs were quantitated as described in Figure 1. [A] For each condition a representative single slice micrograph is shown; arrow heads point out ILEs; scale bar = 30μm. [B] Bar graphs show the means of 3 biological replicates (white circles) ± S.D. Significance was determined as in Figure 1. Total number of analyzed cells per condition: CHIKV 181/25 WT (185), CHIKV 181/25 E2 K200A (167). Please note that the micrograph and data for CHIKV 181/25 WT were reproduced in Figure S2 as experiments were performed in parallel. [C] Location of the E2 K200 residue (pink spheres) in E2 domain B. Crystal structure of the E2-E1 dimer (PDB accession number: 3J2W); E2 and its domains A, B, C, and β-ribbon connector in shades of pink; E1 in grey.

**Figure S5: Human CHIKV patients can produce Abs that attenuate ILE formation.** U-2 OS cells infected with CHIKV 181/25 GFP (MOI_U-2 OS_ 0.25) were incubated with growth medium (no Ab), or growth medium containing a 1:100 dilution of serum (“s”) or plasma (“p”) from either convalescent human CHIKV patient (DC2), a recent Influenza A (IAV) vaccinee, or a control volunteer who was seronegative for both CHIKV and IAV. ILE formation was quantitated at 11 hpi as described in Figure 1. [A] For each condition a representative single slice micrograph is shown; arrow heads point out ILEs; scale bar = 30μm. For clarity, micrographs were pseudo-coloured to represent viral proteins in red and β-tubulin in green. [B] Bar graph shows the means of 3 biological replicates (white circles) ± S.D. Significance was determined as in Figure 1. Please note, subsets of mock and no Ab data were reproduced in Figure 5C, Figure 3C, and Figure 8B as respective experiments were done in parallel. Total number of analyzed cells per condition: mock (230), no Ab (234), control serum (235), DC2 serum (338), IAV plasma (188), DC2 plasma (290).

**Figure S6: Critical binding residues of anti-CHIKV E1 and E2 mAbs in the context of the CHIKV E2/E1 trimeric spike.**[A-B] Crystal structure of the CHIKV E2-E1 trimeric spike (PDB accession number: 3J2W^49^): each E2 protein is shown in a different shade of red; E1 proteins in shades of blue. Highlighted are the critical epitope binding residues for the tested anti-CHIKV E1 and E2 mAbs (as determined by neutralization escape mutants, mutational scanning, or structural studies, Table S1). On the left are the residues for mAbs that attenuated ILE formation (cyan); residues in light green were shared between ILE-attenuating and non-attenuating mAbs. On the right are residues for mAbs that did not attenuate ILE formation (yellow). [A] Sideview of the E2-E1 trimeric spike in relation to the plasma- or viral membrane. [B] Top down view of the E2-E1 trimeric spike along its quasi 3-fold axis
