# Supplementary material for: The alphavirus determinants of intercellular long extension formation
Source: mBio. 2024 Dec 19;16(2):e01986-24. doi: 10.1128/mbio.01986-24 (PMC11796390; doi:10.1128/mbio.01986-24)
Supplement: Table S1 — Characteristics of anti-CHIKV antibodies used in this study. [file mbio.01986-24-s0008.docx]

| **CHIKV target** | **mAb name** | **Epitope domain and key residues** | **Ab binding comments** | **Neutralizing activity on indicated virus IC_50_**^‡^ | **Reactivity to CHIKV 181/25 GFP-infected**  **U-2 OS cells EC_50_^◊^** | **Attenuated infection step** | **Effect on ILEs in U-2 OS cells (this paper)** | **References** |
| --- | --- | --- | --- | --- | --- | --- | --- | --- |
| **E1** | DC2.112°^,3^ | DII  E1: W89^a^, G91^a^, N100^a^ | Binds CHIKV VLP at pH5.5, not pH7.0 | CHIKV 181/25^‡^  72 nM | Strong  0.011 μg/ml | Budding (IgG, Fab) | None (IgG) | (Quiroz et al. 2019; Kim et al. 2021) |
|  | chCHK-166*^,1^ | DII  E1: K52^a^, K61^b^, G64^b^ | Competes with DC2.112 for binding | CHIKV 181/25^‡^  m-IgG: 0.15 µg/ml  CHIKV-RSU1^‡^  m-IgG: 0.04 µg/ml | Strong  0.005 μg/ml | Attachment Fusion  Budding | Inhibits (IgG) | (Pal et al. 2013; Yin et al. 2023; Jin et al. 2015; Kim et al. 2021) |
|  | E10-18*^,4^ | DIII  E1: E302^bc^, L319^c^, Q351^bc^ | Allosterically prevents binding of D3-62, K9-1 to E2 domain A (reverse not true) | CHIKV 181/25 GFP^‡^  No (at 20µg/ml) | Moderate  0.233 μg/ml | n.a. | Inhibits (IgG)  Inhibits (scFv) | Thérèse Couderc, Marc Lecuit and Felix Rey unpublished; (Yin et al. 2023) |
|  |  |  |  |  |  |  |  |  |
| **E2** | chCHK-152*^,1^ | A+B  E2: A11^c^, D59^bc^,M74^c^, N193^c^, G194^c^, T212^c^, N231^b^, H232^c^, K233^b^, W235^c^ | Fab binds on E2 domain A and B, and β-ribbon connector (within single E2) | CHIKV LR2006 OPY1^‡^  0.04 nM  CHIKV 181/25^‡^  0.003 µg/ml  CHIKV 181/25 GFP^§^  0.76 ng/ml | Strong  0.004 μg/ml | Fusion  Budding | None (IgG) | (Pal et al. 2013; Sun et al. 2013; Jin et al. 2018; Quiroz et al. 2019; Yin et al. 2023) |
|  | C9^pMAZ^°^,2^ | A+A’B’  E2: W64^bc^, C91^c^, T92^c^, I93^c^, T94^c^, G95^b^, T155^c^, V157^c^, Q158^c^, S159^c^, T160^c^, A162^ac^, V264^c^, R267^c^  E2’: E24 ^c^, H26 ^c^, S27 ^c^, C28 ^c^, D71 ^c^, N72 ^c^, R119 ^c^, I121 ^c^, T179 ^c^, S182 ^c^, Q183 ^c^, Q184 ^c^, S185 ^c^, G186 ^c^, N187 ^c^, T191 ^c^, N193 ^c^, N218 ^c^, N219 ^c^ | IgG: crosslinks two E2 from different spikes^c^  Fab: binds across two E2 within same spike: domain A and β-ribbon in one, and A and B domain in adjacent E2^c^ | CHIKV 181/25^‡^  0.05 nM | Moderate 0.631 μg/ml | Fusion (IgG, Fab), Budding (IgG, not Fab) | None (IgG) | (Selvarajah et al. 2013; Jin et al. 2015; Jin et al. 2018; Chmielewski et al. 2022; Quiroz et al. 2019) |
|  | ch-m242* | A  K10^c^, A11^c^, T58^c^, D59^c^, H232^c^, K233^c^, W235^c^ | Fab binding on Domain A nudges E2 domain B sideways | CHIKV-LR^‡^  m-IgG: 0.3 µg/ml,  m-Fab: > 20 µg/ml  ch-IgG: n.a. | Moderate  1.62 ug/ml | Entry (inferred by CryoEM structure only) | Moderately inhibits (IgG) | (Jin and Simmons 2019; Sun et al. 2013; Akahata and Nabel 2012) |
|  | K9-1*^4^ | A  E2: W64^bc^ | E10-18 competes with K9-1 binding. Not vice versa. | n.a. | Strong  0.011 μg/ml | n.a. | Inhibits (IgG)  Inhibits (Fab) | Thérèse Couderc, Marc Lecuit, Felix Rey unpublished |
|  | D3-62*^4^ | A  E2: W64^bc^ | E10-18 competes with D3-62 binding. Not vice versa. | n. a. | Strong 0.004μg/ml | n.a. | Inhibits (IgG)  Inhibits (Fab) | Thérèse Couderc, Marc Lecuit, Felix Rey unpublished |
|  | chCHK-265*^,1^ | B  E2: Q183^ac^, Q184^bcd^, S185^bc^, G186^bc^, N187^c^, K189^c^, V192^b^, N193^b^, C203^c^, G204^c^, G209^bd^, L210^bd^, V216^c^, N218^bc^, N219^ac^, C220^c^, K221^c^ | Fab binds lateral tip of B domain, almost parallel to membrane. Neither Fab nor IgG contact with domain A, and no crosslinking of domain A with B | CHIKV 181/25^‡^  ch-IgG 0.28 nM  CHIKV-LR^‡^  m-IgG 0.008 µg/ml  CHIKV-RSU1^‡^  m-IgG 0.005 µg/ml | Moderate  2.45 ug/ml | Entry (post-attachment, pre-fusion)  Budding | None (IgG) | (Pal et al. 2013; Fox et al. 2015; Malonis et al. 2021; Kim et al. 2021; Raju et al. 2023) |
|  | DC2.M108°^,3^ | B  E2: G209^b^, K215^b^ | Competes with CHK-265 but suggested to bind distinct, more lateral epitope | CHIKV 181/25^‡^  0.82 nM | Strong  0.004 μg/ml | n.a. | None (IgG) | (Malonis et al. 2021) |

**Table S1** Characteristics of anti-CHIKV antibodies used in this study.

mAb name: * mAb originally identified in mice, ° mAb originally identified in humans

^1^ Immunogen: natural CHIKV infection (LaRéunion 2006 OPY1, IOL), boosted with either CHIK virus, CHIK VLP, or recombinant CHIKV E2,

^2^ Immunogen: natural CHIKV infection (2007 Romagna, Italy outbreak, IOL),

^3^ Immunogen: natural CHIKV infection (strain not confirmed),

^4^ Immunogen: recombinant, soluble p62-E1 (derived from CHIKV clinical isolate 05-115)

Key residues as identified by: ^a^ Arginine- or alanine-scanning mutagenesis,

^b^ Ab escape mutations

^c^ Cryo-EM or x-ray structure, with or without Proteins, Interfaces, Structures, and Assemblies (PISA) solvent exclusion analysis

^d^ ELISA binding

Neutralizing activity on : ^‡^ FRNT on Vero cells, ^§^ FRNT on MEF cells

^◊^ FigS2 / S5; IgG in fixed cell surface ELISA with CHIKV 181/25 GFP, EC_50_ are indicated

n.a. not available; m-IgG mouse IgG; ch-IgG chimeric IgG
